# Integrating computational and experimental approaches to identify antigen–specific antibodies

**DOI:** 10.3389/fimmu.2026.1951538

**Published:** 2026-10-29

**Authors:** Maria Francesca Abbate, Paul Pereira, Pierre Toxe, Nicolas Maestrali, Marie Gagnaire, Emmanuelle Vigne, Melody A. Shahsavarian, Thierry Mora, Aleksandra M. Walczak

**Affiliations:** 1 Laboratoire de physique de l’École normale supérieure, CNRS, PSL University, Sorbonne Université, and Université de Paris, Paris, France; 2 Large Molecule Research, Sanofi, Vitry-sur-Seine, France

**Keywords:** antibody repertoires, antigen specific antibody, computational analysis, immune repertoire sequencing, single cell sequencing

## Abstract

The identification of therapeutic antibodies is critical for developing effective treatments for many diseases. We present a joint computational and experimental approach for identifying antigen–specific antibodies. We develop and test our pipeline on a probe antigen: GFRAL fused with Diphtheria Toxin Fragment A (DTA). Using humanized Trianni mice, we conducted a longitudinal study with repeated blood sampling and splenic analysis. We applied the STAR computational method that exploits similarity network properties on bulk antibody repertoire data sampled at key time points. By mapping the output from STAR to single cell data taken at the last time point, we successfully identified a pool of antibodies, of which 45% demonstrated binding capabilities. We observed convergent selection, where responding sequences with identical amino acid complementarity determining regions 3 (CDR3) were found in different mice. We provide a catalog of 70 experimentally validated antibodies against the GFRAL-DTA fusion antigen. This study underscores the utility of integrating computational methods and experimental validation for antibody discovery in therapeutic contexts by reducing time and increasing efficiency.

## 1 Introduction

The discovery of novel therapeutic antibodies has revolutionized the treatment of various diseases (1–4), particularly in the fields of oncology (5, 6), immunology (7), chronic diseases like migraines (8), and metabolic disorders (9, 10). Antibody-based therapies have enabled targeted treatments that minimize off-target effects compared to traditional drugs (11). Antibody discovery relies on three main approaches: *in vivo*, *in vitro*, and in silico (12). *In vivo* methods recover antigen-specific antibodies from the B cell repertoires of immunized animals, often achieving very high affinities in the picomolar range, but are often time– and resource–intensive (13–18). *In vitro* methods, such as phage display, enable screening of large natural or synthetic human antibody libraries as ready-to-use repertoires (19–22). While these methods offer scalability and speed while bypassing animal immunization, they typically yield medium affinities in the nanomolar range due to inherent limited library diversity (23–26). Antibodies derived from both *in vitro* and *in vivo* approaches often require optimization to achieve desired binding and functional properties, as well as to address developability liabilities (27–30). *De novo* antibody design has shown recent advances, albeit with limited success rate (31). More broadly, recombinant display methods that also include yeast, bacterial, and mammalian display have been used in antibody discovery (32, 33), with yeast display routinely used for antibody engineering. However, methods like mammalian display, which are closest to the desired biophysical properties, are limited in throughput (34, 35). Currently, in silico methods use computational tools to predict and refine biophysical properties and improve manufacturability (31, 36–38). They play an increasingly important role in complementing traditional methods and streamlining antibody development. In this study we develop a method that leverages *in vivo* experiments with computational analyses to select specific antibodies ahead of their testing.

Specifically, we focus on strengths of both computational and experimental methods. We designed an immunization and sequencing pipeline by vaccinating Trianni mice and sampling their repertoires using a combination of bulk and single cell methods. Single cell sequencing depth is limited by costs. Bulk repertoire sequencing offers larger depth but does not allow for light-heavy chain pairing. We used bulk repertoire sequencing data to identify candidates and further determined their cognate light chains using single cell data. We then evaluated their binding abilities, achieving a 45% success rate. We analyzed the experimentally validated binders and non-binders, providing sequence level information about the binding properties.

To identify potential binders from bulk repertoire sequencing we used the previously developed STAR method (39). STAR is designed to enhance antibody discovery by analyzing bulk repertoire data without prior knowledge of longitudinal data. Instead it exploits similarity network properties. It identifies statistically significant clusters of similar sequences. The over-representation of sequences with large number of neighbours has been shown to be indicative of potential binders.

We develop our method on a test immunogen, the GDNF family receptor alpha-like (GFRAL) protein (40–43) fused with the Diphtheria Toxin Fragment A (DTA). The GFRAL receptor mediates the effects of the cytokine GDF-15, known for its role in appetite regulation and stress responses (44, 45). As GDF15 is released in response to cellular stress or injury, it serves as a “distress signal” (46), binding to GFRAL to trigger nausea, suppress appetite and reduce food intake (47). The DTA (Diphtheria ToxinA) tag was incorporated to enhance antigen presentation and stimulate a more robust immune response. While GFRAL alone has attracted significant attention due to its potential role in treating conditions related to appetite dysregulation, including cancer cachexia (46, 48, 49), anorexia (47), obesity (50), metabolic diseases and diabetes (9, 51), in the current work the GFRAL-DTA construct is used solely as a probe antigen since we cannot separate the effects of the DTA tag on binding.

We aimed to detect antibodies specific to this probe through a unique approach combining *in vivo* experiments, repertoire computational analysis, and *in vitro* validation. The integration of bulk and single cell data allowed us to identify and validate antibodies against GFRAL-DTA, resulting in a list of 70 binders, including some not observed in the sequencing dataset.

## 2 Results

### 2.1 Experimental and computational workflow identifies candidate GFRAL-DTA-specific antibodies

Developing human antibodies is essential, as non-human antibodies can elicit immune responses, such as the human anti-mouse antibody (HAMA) reaction, which can diminish therapeutic efficacy and increase safety risks (2). To avoid this issue, we worked with Trianni mice (52), which are genetically engineered to produce chimeric antibodies with fully human variable regions (VH,VLκ). A group of 5 Trianni mice were immunized with four shots of injection of a human GFRAL Diphtheria Toxin Fragment A (huGFRAL-DTA) fusion protein, where DTA serves as an adjuvant, taking blood samples four days before and after each injection, and spleen samples from the top three responders after three months. We collected RNA-seq bulk sequences from each time point and paired single cell sequences on day 25, day 46 and day 67 (**Figure 1A**). Only samples from the top three responders were analysed, as is the standard therapeutic program protocol since it is difficult to exploit limited humoral responses.

**Figure 1:**
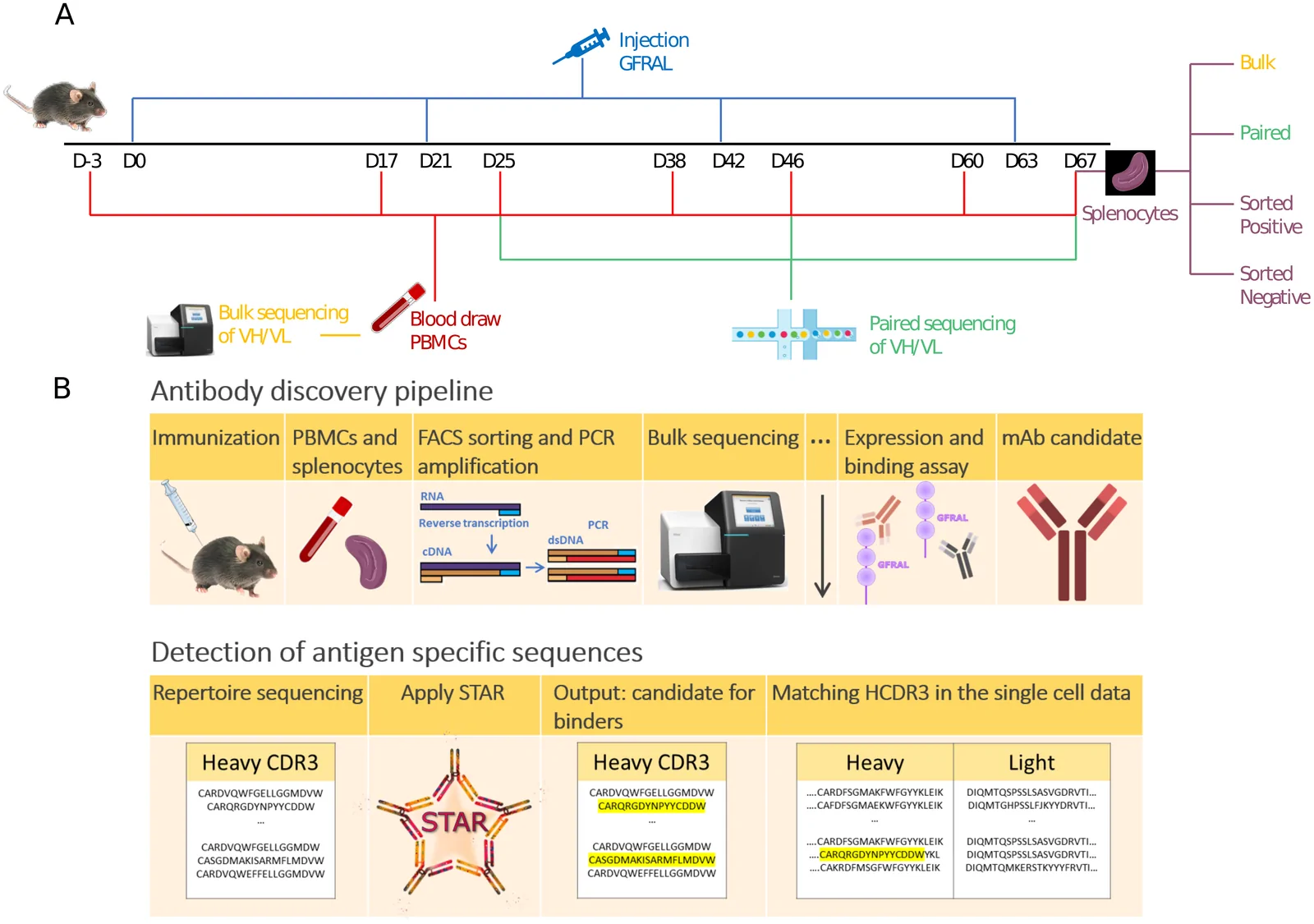
Experimental setup for the immunization and antibody discovery pipeline. **(A)** Trianni mice were immunized with the GFRAL-DTA target at four time points (days 0, 21, 42, and 63). Blood samples were collected on days -3, 17, 25, 38, 46, 60, and 67 to monitor immune responses over time. In addition, spleen samples were obtained from three of the five mice on day 67. Bulk sequencing of both heavy and light chain B cell repertoires was performed for all time points. For select days (25, 46, and 67), a subset of cells were sequenced in single cells to obtain paired heavy and light chains. From the spleen samples on day 67, cells were sorted between GFRAL-positive and GFRAL-negative by flow cytometry, and sequenced in single cells for paired chains. **(B)** Overview of the antibody discovery pipeline using STAR to detect the antigen specific sequences. The process begins with immunization with the GFRAL-DTA target, followed by isolation of PBMCs and splenocytes and FACS sorting to enrich for B cells. After PCR amplification and sequencing, we applied the computational method STAR (39) to each sample after the first injection, using only the heavy CDR3 repertoire. The identified sequences were matched to the results of the single cell sequencing experiments to reveal their corresponding light chain, when found. The selected candidates were expressed and tested for binding using surface plasmon resonance.

We analyzed the bulk heavy-chain immunoglobulin (IgH) repertoire sequencing data from blood samples of 3 Trianni mice across different time points: days −3, 17, 25, 38, 46, 60, and 67, with injection on days 0, 21, 42, and 63. We observed a total of 3,160,880 unique nucleotide sequences and 1,530,511 unique amino acid sequences, combining the data at all timepoints and mice (see **Supplementary Table S1**). Additionally, to obtain reliable frequency estimates and chain pairing, we incorporated single cell sequencing data from blood samples on days 25, 46, and 67, with a total of 11,489 paired sequences, and spleen samples on day 67, which were sorted into antigen-positive and antigen-negative cells, using cynoGFRAL without the DTA domain, resulting in a total of 22,898 successfully paired sequences (**Figure 1B**).

The overlap between the different datasets (blood vs spleen, single cell vs bulk, antigen-positive vs negative and binders) are summarized in **Figure 2**. Overall, overlap is low because many sequences are rare and seen just once (**Figure 2A**). However, we observed that the single cell sequences from the spleen were largely included in the corresponding bulk spleen data, whereas the bulk blood repertoire captured only a limited portion of the single cell blood data (**Figure 2B**). This indicates that, for blood samples, it is beneficial to integrate both bulk and single cell sequencing to maximize the recovered information.

**Figure 2:**
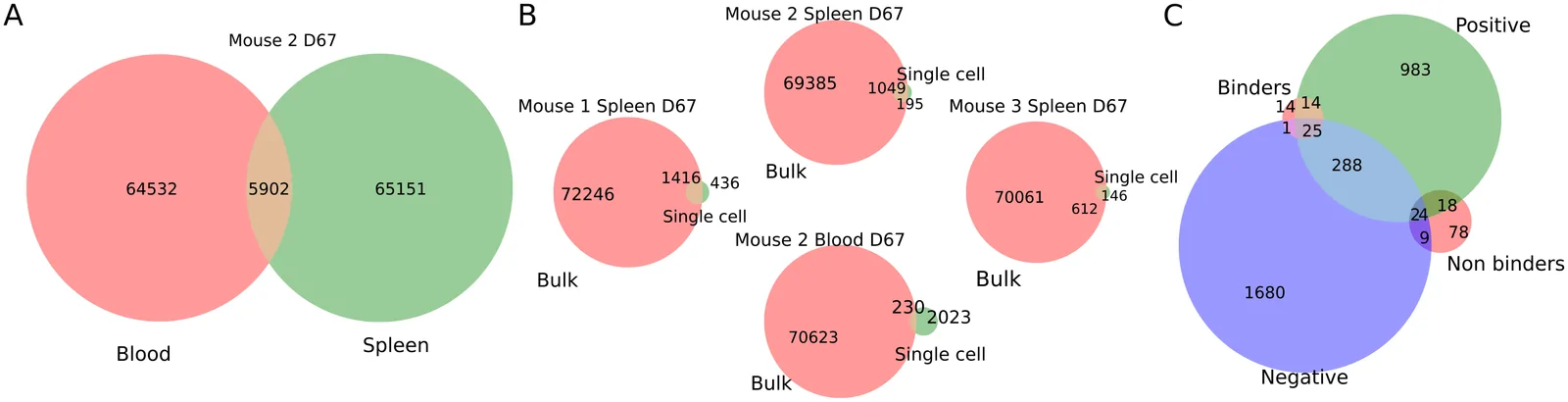
Repertoire overlap. **(A)** Overlap between blood and spleen bulk repertoires for mouse 2 at day 67. **(B)** Comparison of the overlap between bulk and single cell sequences from the same tissue and day (D67). Specifically, it includes mouse 1 spleen (bulk vs. single cell), mouse 2 (spleen and blood) bulk vs. single cell, and mouse 3 spleen (bulk vs. single cell). This comparison indicates that most information from the single cell spleen data is captured by the corresponding bulk spleen data, whereas the bulk blood data covers only a limited portion of the single cell blood repertoire. **(C)** Overlap between SPR-validated binding and non-binding sequences versus sequences sorted as antigen-positive and non-positive. Data from all mice. A description of the SPR experiments can be found in the**”**Surface Plasmon Resonance Binding**”** section of Material and methods.

In the primary workflow, we treat each bulk data time point as independent and analyze them using the STAR method (39), which looks for signatures of affinity maturation as clumps of closely related sequences in repertoires. Briefly, the method identifies clusters of sequences with statistically overrepresented numbers of neighbors as potential responders. Neighbors are defined as unique CDR3 nucleotide sequences differing by one amino acid. We first cluster heavy-chain CDR3 sequences from bulk repertoire sequencing using this definition of neighbors, and associate each sequence to the cluster it belongs to. For each sequence, we then compute the number of neighboring sequences within the sample (**Figure 3**). Sequences that have a sufficient number of neighbors, determined by a threshold of 300 neighbours set using the STAR method (**Figure 3A**), are pre-selected. These sequences are then determined as potential responders (hits), if they belong to a cluster that contains at least 10 such sequences, a parameter that is optimized by STAR. The number of hits identified per mouse and time point is shown in **Figure 3B**. This approach ensures that only clusters with substantial neighbor relationships are considered, helping to exclude isolated clones which, despite having numerous neighbors, do not form dense clusters but rather more linear ones.

**Figure 3:**
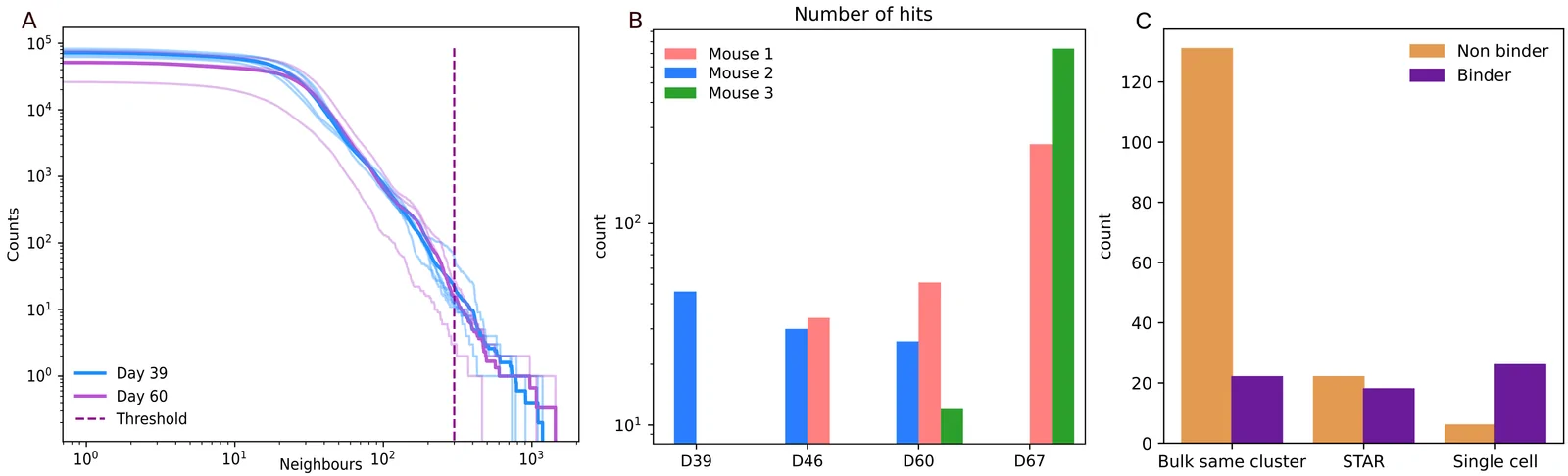
Number of neighbors, hits and experimental results. **(A)** Distribution of number of neighbors for each mouse at day 39 and at day 60. Thick lines show the mean over mice (N = 3). According to the STAR method, sequences above the threshold of 300 neighbours are identified as hits if their cluster contains at least 10 sequences that also exceed the threshold. **(B)** Number of hits identified by STAR for each mouse and day from bulk data. **(C)** Experimental results by categories: number of experimentally tested sequences identified as binders/non binders by SPR across different categories: the output of STAR, sequences from the bulk repertoire belonging to the same clusters of the sequences as output of STAR and sequences with high frequency in the single cell dataset. Of the 40 sequences output by the STAR pipeline, 18 were validated as binders. Additionally, testing of sequences from the single cell dataset based on frequency yielded 26 binders, while 22 binders were identified from bulk repertoire sequences that were absent in the single cell data, and 4 binders were found in the randomly tested single cell sequences, expanding the pool of potential binding antibodies.

Next, we matched these hits with the single cell sequencing data to identify the corresponding light chains (**Figure 1B**). For each cluster, we selected a representative sequence with the highest number of neighbors to serve as the reference for matching in the single cell dataset. We found that 40 of the sequences identified through STAR were present in the single cell dataset, and we successfully paired their heavy chains with light chains, showing little sequence level similarity between them (**Supplementary Figure S1**).

### 2.2 Validation of GFRAL-DTA specificity by surface plasmon resonance

We then expressed these sequences as IgGs and assessed their binding to the huGFRAL-DTA immunogen using Surface Plasmon Resonance (SPR) (SI Dataset 1, see “Surface Plasmon Resonance Binding” section of Material and methods for details). Of the 40 proposed sequences, 18 were successfully validated as binders by SPR, with 5 demonstrating subnanomolar affinity (**Figure 3C**, STAR bars) and **Supplementary Figure S5**. Given that these 40 sequences were drawn from a total population of 1,530,511 sequences from bulk sequencing, the probability of finding 18 binders in this group is very low, especially considering that they were required to match sequences from the single cell dataset.

This two-step approach—combining deep bulk repertoire sequencing with single cell sequencing—enables us to leverage the depth of bulk sequencing and the chain-pairing precision of single cell sequencing, thus mitigating the limitations of each method. Single cell sequencing provides much lower depth than bulk sequencing, which limits its ability to capture rare BCRs and to assess the level of clustering, while bulk sequencing lacks paired chain information.

In addition to the STAR hits, we selected more antibody sequences to be tested by SPR based on two additional criteria: (1) the most frequent sequences from non-sorted single cell data, independent of bulk data (32 sequences), which showed an 80% success rate, indicating that frequency after a certain threshold in single cell data is a good predictor for binders (**Figure 3C**, Single cell bars); (2) randomly selected 20 antibodies from the non-sorted single cell dataset, which yielded a much lower binding success rate of 20%; and (3) reconstructed antibodies with heavy chains from the same bulk cluster as the initial 40 hits, but with different mutation levels and frequencies in the heavy CDR3 region, paired with the corresponding light chain from single cell data 154 sequences. Specifically, for each of the 40 sequences output of STAR belonging to 40 different clusters, we took 3 or 4 sequences from these clusters, with high, medium and low bulk frequencies and mutation levels compared to the template sequence. This yielded sequences that were not present in the single cell dataset, which gave a 14% binding success rate (**Figure 3C**, Bulk same cluster). Considering that most sequences in this group were absent from the single cell dataset, this 14% success rate (corresponding to 22 sequences) has far greater significance compared to the 20% rate for random single cell sequences selected from the non-sorted single cell dataset (corresponding to 4 sequences). This finding demonstrates that pairing bulk-derived heavy chains with corresponding light chains from the single cell data can yield functional, target-specific antibodies not originally found in the single cell dataset. Consequently, this strategy expands the pool of potential candidates for monoclonal antibody development by generating viable binders beyond those directly observed in the single cell data. We tested 251 sequences total, with another 5 additional negative and positive controls (see SI Dataset 1).

The overlap between SPR validated binding and non-binding sequences, versus those sorted as positive and non-positive (**Figure 2C**), shows that being positive is not a good predictor of binding. However, there is a slight enrichment of non-binders among the negative-only fraction (9 non-binders out of 10 tested that were sorted negative vs 51 non-binders among 91 sorted as positive or negative). The sorting is not entirely consistent with the SPR assay: some binders are found in the negative sorted set. Also, there is a large overlap between the positive and negative fractions. This is likely due to low B cell receptor expression levels in some cells, which may have led them to be sorted into the negative fraction despite carrying antigen-specific sequences, as well as the experimental conditions used for sorting (antigen concentration, incubation time, washes, etc). For this reason, we consider these sequences present both in the negative and positive sorts to be likely antigen-specific candidates.

### 2.3 Origin and lineage structure of GFRAL-DTA antibodies

We investigated the origin, distribution, abundance, and shared characteristics of sequences experimentally validated as binders with SPR. **Figure 4A** shows the sequence counts of the binders color-coded by origin (blood, spleen, mouse, day, and synthetic sequences that were absent from the single cell dataset, shown in grey). This plot visualizes the primary sources of the binding response, indicating that, apart from mouse 1 where binders predominantly originated from spleen samples, the other mice show relatively balanced contributions from both blood and spleen samples.

**Figure 4:**
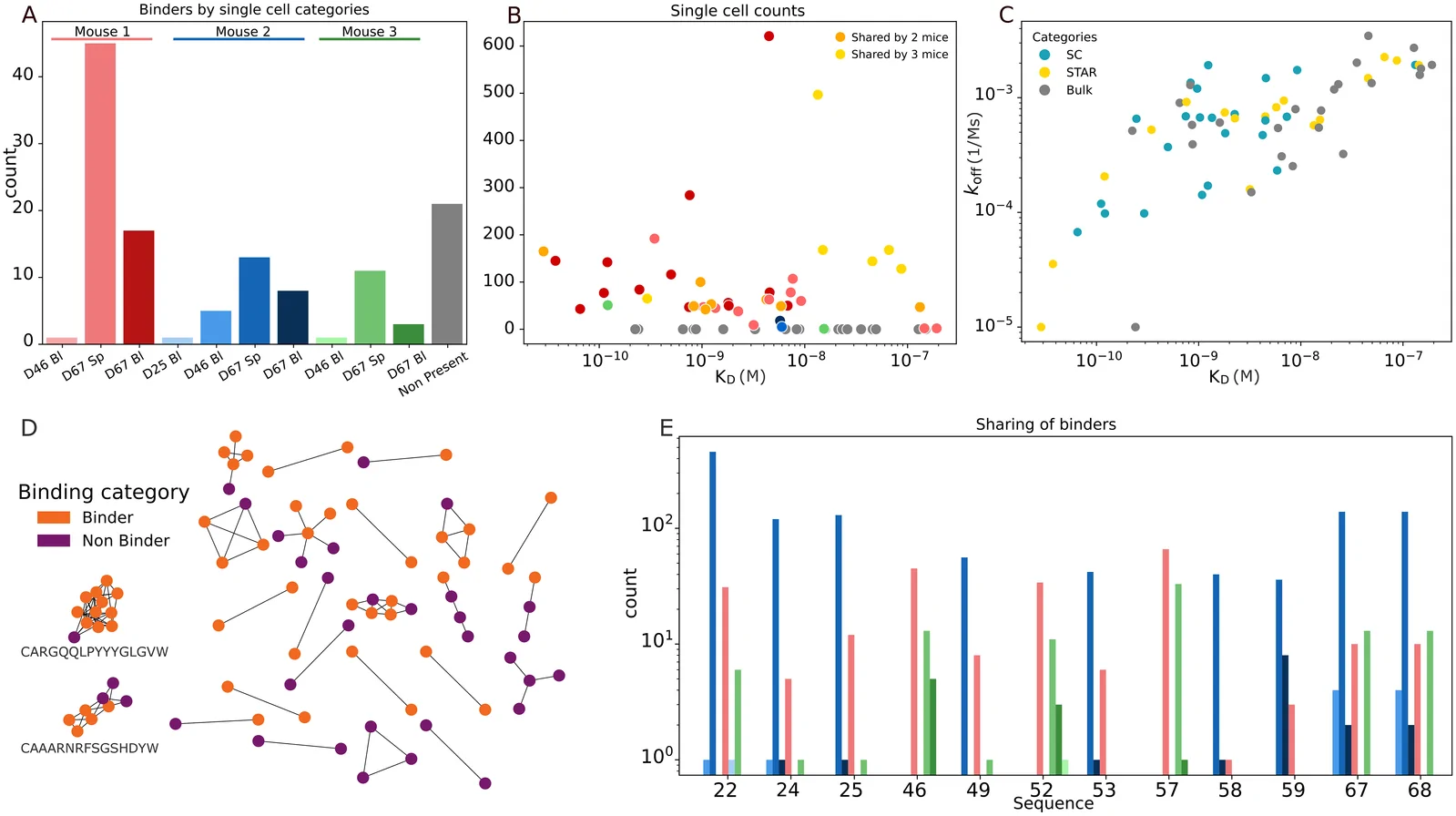
Single cell analysis of binders by sample type, sequence abundance, and cross-mouse sharing. **(A)** Counts of validated binder sequences in the single cell dataset. Each color represents a mouse: red – mouse 1, blue – mouse 2, green – mouse 3. Different hues of each color represent different tissue sources (blood = Bl or spleen = Sp) and days for that mouse. Grey bar (22 sequences) indicates synthetic sequences that were not present in the single cell data, which means they were identified only by STAR, not by the single cell criterium. They correspond to bulk sequences belonging to STAR clusters. Binder sequences are generally distributed across blood and spleen, with a distinct bias towards spleen-originating sequences in mouse 1. B and C Scatter plot comparing the dissociation constant (K_D_) values of binders with their counts in the single cell dataset **(B)** and their dissociation rate k_off_ as measured by surface plasmon resonance (SPR). Grey points were not present in the single cell data and correspond to bulk sequences belonging to STAR clusters. **(C)** Results indicate no correlation between K_D_ and single cell abundance but a strong correlation (Spearman ρ = 0.67)) between K_D_ and the dissociation rate. Strong binders (lower K_D_) may have high or low representation, including those missing from single cell data (grey points). The colors identify if the sequences were selected using the pipeline STAR (yellow) or according to other methods: most frequent in single cell SC (cyan) or bulk sequences belonging to STAR clusters (grey). Similarity network between antibodies tested for their binding properties using SPR. Clusters with only one antibody sequence (177 antibodies) are not shown. Each node represents heavy-chain CDR3 sequences, colored as binder (orange) or non-binder (purple), with edges connecting sequences differing by one amino acid. Consensus sequences for the two biggest clusters are provided. **(E)** Cross-mouse sharing of SPR-validated binder sequences. Out of 70 validated SPR binders, 12 were shared between different mice. For each of these 12 sequences listed on the x axis, the bars show abundances in each tissue and day sample [same color code as **(A)**]. Shared binders suggest convergent selection across mice and tissues. 6 out of 12 binding sequences are shared by all mice and 6 are shared between two mice.

**Figure 4B** compares each binder’s dissociation constant *K_D_* to its abundance in the single cell data, revealing only a weak correlation between them (Spearman *ρ* = 0.26, *p* = 0.03). Strong binders are found across sequences with varying counts in the single cell data, including those entirely absent from it (grey points), underscoring that affinity is not well predicted by abundance in single cell data. We next examined the relationship between the dissociation and equilibrium rate constants *k*_off_ and *K_D_* obtained from the SPR measurements (**Figure 4C**, see SI Dataset 1 for raw data files and **Supplementary Figure S5** for SPR sensograms and **Supplementary Figure S2** for organs and mice the sequences originate from). The scatter plot shows that antibodies with lower *K_D_* values (stronger affinity) generally display lower *k*_off_ values, marking slower dissociation, consistent with tighter binding (Spearman *ρ* = 0.67). Additionally, 7 newly tested antibodies display very strong binding with *k*_off_ ≤ 10^−4^ *s*^−1^ (**Supplementary Dataset S1**, which shows 9 such antibodies including two positive controls). Antibodies with high *K_D_* and small *k*_off_ come from candidates identified by all three methods: STAR, frequent single cells and bulk sequences belonging to the same STAR clusters (**Figure 4C**).

To visualize how similar the SPR-validated antibodies are at the sequence level, we built a network representation of all SPR tested antibodies, where each node corresponds to a heavy-chain CDR3 sequence and edges connect sequences differing by one amino acid (**Figure 4D**). Since we are interested in similarity networks between binders, we do not show clusters made out of only one antibody. Nodes are colored according to their experimental classification as binder or non-binder. We observe a significant overrepresentation of connected nodes sharing the same label (binder/non-binder) than expected by chance (74% vs 49% expected by chance, *p* = 3.86×10^−8^, binomial test). **Figure 4E** shows the abundances in each mouse of the 12 SPR-validated binders that were shared between different mice. 6 out of 12 binding sequences are shared by all mice and 6 are shared between two mice, suggesting convergent selection in the immune response.

To evaluate shared response patterns during affinity maturation, we reconstructed lineages of antibody sequences from the splenocyte single cell data sorted into antigen-positive and antigen-negative cells for lineages shared by three mice (**Supplementary Figure S3**). Lineages were inferred using the HILARy software (53), which incorporates heavy and light chain pairing, V and J gene usage, and CDR3 length. Phylogenetic rooted trees were generated with RAxML (54), assigning the inferred germline as the root, and visualized in iTOL (55). The lineage reconstruction revealed that, in each mouse, the largest lineages were predominantly composed of antigen-positive sequences (**Supplementary Figure S4**). This observation is consistent with large lineages originating from the process of affinity maturation, which results in high-affinity binders. To compare lineages across mice, we clustered the CDR3 amino acid sequences of all mice together using single-linkage clustering with links between any pair of sequences differing by at most one amino acid. While the largest lineages were private to individual mice, meaning that sequences of those lineages did not cluster with any other from other mice, we observed medium-sized lineages containing antigen-positive sequences that formed clusters across all three mice (same color in **Supplementary Figure S3**). This suggests that different ancestral cells may have independently acquired mutations leading to a shared immune response across mice. STAR identified hits span all mice, trees and lineages (asterisk in **Supplementary Figure S3**).

### 2.4 AlphaFold 3 analysis of experimentally validated antibody sequences

To explore the structural basis of antigen-antibody binding, we modelled 137 of the experimentally tested antibodies using AlphaFold 3 (56). We acknowledge that this analysis should be interpreted cautiously because antigen-antibody interface confidence in structure prediction models is generally quite low. Antibodies were categorized based on their experimental binding affinities, determined through surface plasmon resonance (SPR) assays, into 70 binders and 67 non-binders. For each sequence (heavy and light chain) along with its target antigen, we used AlphaFold 3 to predict the folding and binding structure (**Figure 5A** for examples from each binding category) and to produce the interface predicted aligned error (ipTM), a metric that estimates the confidence of the predicted inter-residue interactions at the binding interface. ipTM values range from 0 to 1, where higher values indicate a greater likelihood of a correct structural alignment and potential interaction between the antigen and antibody chains.

**Figure 5:**
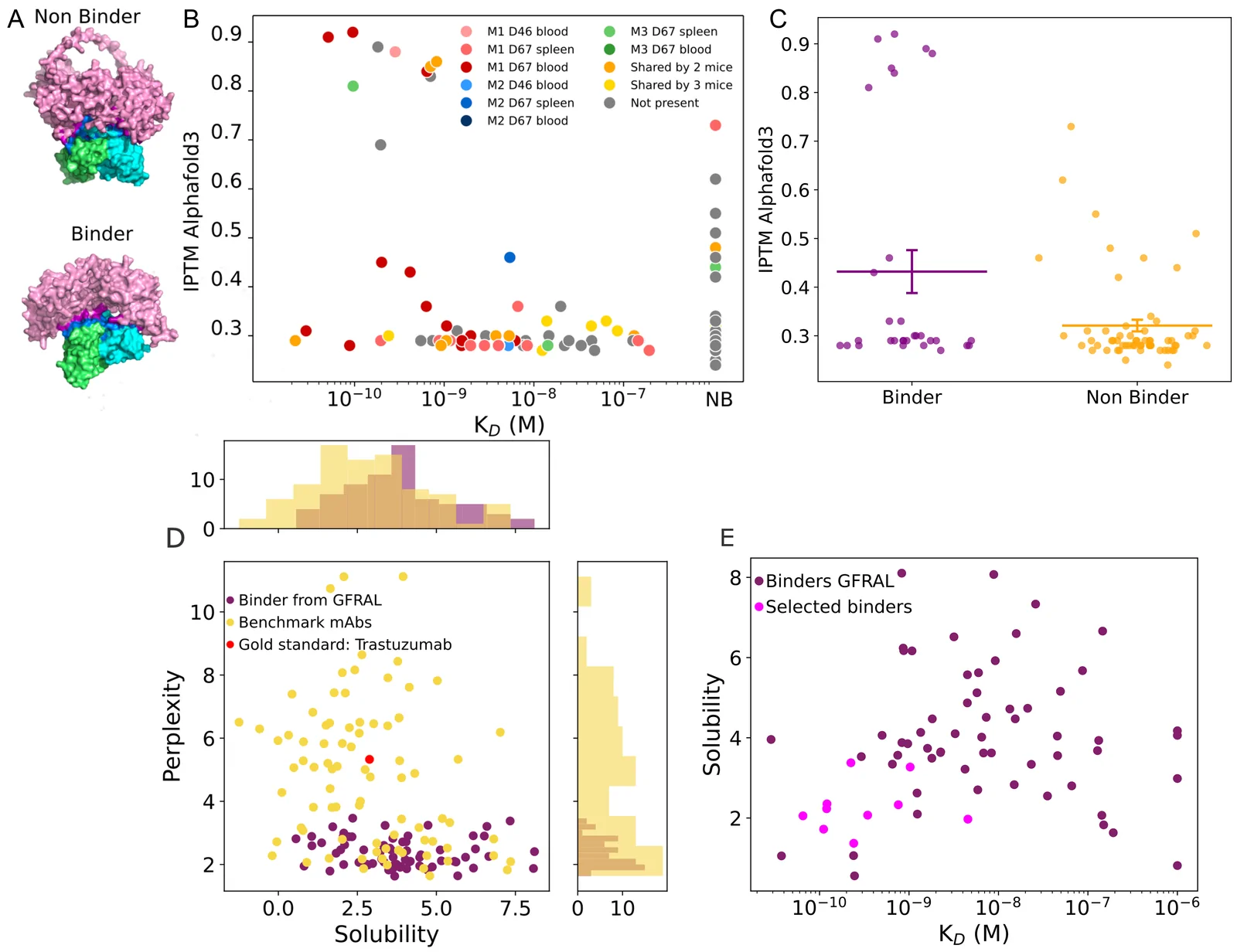
Predicted binding strength by AlphaFold 3. A. Examples of predicted structures from AlphaFold 3, plotted using PyMol (57), for three antibody-antigen complexes from each binding category. The antigen is represented in pink, the light chain in green and the heavy chain in cyan. The interface where residues from different proteins are in contact is shown in purple for the antigen (epitope) and in blue for the antibody (paratope). The strong binder shows a more compact interaction with the antigen, while the non-binders exhibit more spread-out configurations. This compact structure in the strong binder is consistent with its higher AlphaFold 3 ipTM score, supporting the ipTM as a proxy for binding affinity. **(B)** Scatter plot of AlphaFold 3 ipTM scores vs. experimental binding affinity (K_D_) from SPR. Colors show the origin of sample. The plot indicates a moderate negative correlation between AlphaFold 3 ipTM scores for K_D_ (Pearson ρ = −0.48, p = 10^−5^), suggesting that higher ipTM values may be associated with stronger binding. **(C)** The AlphaFold 3 ipTM scores by binding category along with the mean for each category and the standard error on the mean. The results confirm that good binders have on average a higher AlphaFold 3 ipTM score. Computationally predicted solubility and humanness (perplexity) scores for SPR-validated GFRAL-DTA binders (purple dots), for a reference set of monoclonal antibodies currently in clinical trials (48) (yellow dots) and the industry golden standard Trastuzumab. Scores are calculated as described in Materials and Methods section 4.5 and 4.6. The predicted perplexity for the antibody is calculated as the product of the perplexities of the light and heavy chains. Top histogram: distribution of predicted solubility scores; right histogram: distribution of perplexity scores. SPR-validated GFRAL-DTA binders span a similar solubility range and have lower perplexity scores than reference monoclonal antibodies. **(E)** Computationally predicted solubility score for SPR-validated GFRAL-DTA binders shows a weak correlation with affinity K_D_ (Spearman ρ = 0.21, p = 0.013). Ten predicted binders (pink) were chosen for additional kinetic validation, based on the following criteria: highest binding affinity, maximum sequence diversity, and absence of critical liability factors (free cysteines or N-glycosylation sites).

The results are summarized in **Figure 5B**, which compares the ipTM values between the antibody and the antigen to the measured *K_D_* of all antibodies tested by SPR and shows the average ipTM scores for each binding category. Although most sequences show relatively low ipTM scores (suggesting uncertainty or incorrect predictions at the binding interface), binders tend to have higher average ipTM values compared to non-binders (**Figure 5C**). This indicates that, while AlphaFold 3 is not yet fully reliable in modeling the binding interface (average ipTM ~0.3), and in distinguishing between binders and non-binders, it may still provide valuable structural insights, particularly when the ipTM score is significantly high.

In contrast, AlphaFold 3 consistently showed high confidence in the relative orientation of the heavy and light chains of the antibody itself, with an average inter-chain ipTM ~0.8 for heavy-light chain interactions. This suggests that while AlphaFold 3 can model the overall antibody structure fairly well, it poorly captures its interaction with the antigen. This observation aligns with recent studies (58), highlighting the complexity of modeling antibody-antigen interactions and the need for enhanced algorithms specifically tuned for inter-protein docking predictions.

### 2.5 Experimental validation of the biophysical properties

To assess whether the identified antibodies also possess suitable biophysical properties for therapeutic development, we computed their perplexity and solubility scores following the method described in Materials and Methods sections 4.5 and 4.6, following Pereira et al. (37). Lower perplexity and higher solubility correspond to more favorable developability profiles. In **Figure 5D**, these two scores for the SPR-validated GFRAL-DTA binders are compared to a reference set of monoclonal antibodies currently in clinical trials (48). Despite a slight rightward shift in the GFRAL-DTA distribution, indicating marginally higher predicted solubility scores, the two sets have largely overlap in solubility and the SPR-validated GFRAL-DTA binders rank on the low end of the perplexity distribution, suggesting that the GFRAL-DTA antibodies exhibit developability comparable to clinically advanced molecules. This result is expected since Trianni mice generate human antibodies and validates the accuracy of the perplexity scores. In **Figure 5E**, focusing only on the SPR-validated GFRAL-DTA binders, we observe a modest correlation between solubility score and binding affinity (*K_D_*) (Spearman *ρ* = 0.21, *p* = 0.013). Of note, 35 of the SPR tested antibodies perform better in terms of computational humanness and solubility scores than the current golden standard, Trastuzumab. While these computational methods cannot replace experimental validation, together, these computational assessments indicate that several GFRAL-DTA binders combine strong affinity with promising developability characteristics.

Finally, to experimentally evaluate the pipeline’s ability to identify antibody sequences without developability issues, we selected the top 10 clones based on three criteria: highest binding affinity, maximum sequence diversity, and absence of critical liability factors (free cysteines or N-glycosylation sites). These antibodies were produced and purified, and their biophysical properties were characterized using Trastuzumab as a reference control. We applied multiple orthogonal analytical techniques—size exclusion chromatography (SEC), hydrophobic interaction chromatography (HIC), analytical cation exchange chromatography (aCEX), and capillary electrophoresis SDS (cE-SDS) under reducing and non-reducing conditions—to assess key quality attributes (**Supplementary Figure S6**). This panel enabled detection of fragments, multimers, and aggregates, as well as evaluation of hydrophobic heterogeneity, charge variants, and structural integrity. Nine of the ten antibodies exhibited quality profiles that met or exceeded our predefined standards across all parameters. The experimental assays further validate that the combined computational and theoretical pipeline identifies antibody candidates with good developability characteristics.

## 3 Discussion

In this study, we demonstrated a joint computational-experimental approach for identifying antigen-specific antibodies from mice immunized with a GFRAL-DTA fusion, which we validated experimentally with promising results. Specifically, we showed how using computational methods and exploiting similarity networks can help identify potential antibodies targeting an antigen of interest. Our work also provides a list of 70 monoclonal antibodies (mAbs) that were validated to bind to the immunogen, with the top 10 showing promising developability features.

However, our current approach has several limitations that warrant future improvements. One major challenge is the need for single cell data to pair heavy and light chains. While we successfully mapped the heavy chains predicted by our bulk data analysis to the corresponding light chains using single cell data, this reliance on single cell information restricts the scalability of the pipeline. An important next step would be to develop a purely computational method to predict these pairings, possibly through machine learning (ML) models that can infer the likely pairing of heavy and light chains based on sequence features. Alternatively, novel bulk sequencing methods that capture both chains such as TIRTL-seq (59) would overcome the scalability issue. A given CDR3 heavy chain may be paired with multiple heavy and light chains. Given the depth of single cell sequencing most of our single cell sequences revealed a unique pairing but in case of multiple pairing deeper statistical analysis is needed to choose the best candidate. However, given current single cell sequencing costs, the presented matching approach still offers advantages over both bulk and single cell sequencing. While the need and benefits of combining the two approaches are clear, it is not clear how to do it in practice, since frequency of occurring in the bulk repertoire is not necessarily informative of response (39). Our approach provides a way of combining single cell and bulk sequencing method that exploits both their strengths. It also allows for identifying less frequent binders that were not captured by the single cell assay (gray bar in **Figure 4A**). The strongest binders come from candidates identified by all three assays: STAR, frequent cell and bulk sequences belonging to STAR clusters (**Figure 4C**), which shows that combining different approaches is worthwhile and STAR based analyses allow for identification of new strong binders.

While not a general limitation of the method, our experiments did not use unique molecular identifiers (UMIs) in the sequencing protocol (60), which impacts the ability of STAR to reliably call hits. Future experiments should use UMI to achieve the best possible performance.

Another limitation is the complexity of extending the pipeline beyond GFRAL-DTA specific antibodies. While our results are promising, further validation is required to assess the generalizability of the computational pipeline to other targets. Testing this approach on additional targets will help determine how broadly applicable it is in the antibody discovery process. Additionally, improving the statistical models used to match bulk and single cell data would streamline the process and allow for more accurate predictions without relying heavily on experimental validation at each step.

While it would be interesting to analyze how binding clusters split between λ and *κ* light chains, the Trianni mouse strain that was used is humanized for the heavy and *κ* V(D)J genes but not λ, therefore only *κ* genes were sequenced. In addition, mice predominantly utilize the *κ* locus, with a *κ*:λ ratio of approximately 10:1. Investigating the contribution of different light chain isotypes represents an important direction for future studies, particularly in human antibody repertoires.

In terms of the specific antigen presented, our method identifies potential GFRAL-DTA binders. Mice were immunized with a huGFRAL-DTA fusion and the SPR data were generated using the same protein. We used the full immunogen for binding evaluation, to test the validity of the pipeline that identifies antibodies specific for this *in vivo* stimulant. We emphasize that within the current framework we have not shown that the identified antibodies are directed against GFRAL, as they could also recognize the DTA domain. Yet, for technical reasons, we used a cynoGFRAL protein without a DTA domain for sorting the B cells for single cell sequencing. This strategy enriches in human-cyno crossreactive mAbs (cyno and human GFRAL extracellular domains share 94% identity) targeting the GFRAL extracellular domains rather than DTA. The single cell dataset serves as a reference of the hit rate using the traditional workflow and it would not have been significantly different if we had used the huGFRAL-DTA for sorting. As a result, our binders that are also identified as positive in the single cell dataset are more likely to be GFRAL binders.

Our method simplifies the time and costs of the initial steps of antibody discovery. Additional cellular validation is needed before these antibodies can be tested in animal models. Specifically, it does not bypass the cell binding, cell signaling, competition-inhibition assays, as well as epitope mapping for the identified antibodies.

Despite these limitations, the computational pipeline represents a significant advancement in the efficiency and timeline of the antibody discovery process. By identifying antigen-specific sequences from bulk data, we can narrow down potential binders before moving to more labor-intensive single cell and experimental validation. This offers considerable time savings and the potential to scale up discovery efforts.

Additionally to identifying responders, we report relatively modest overlap between sequences sampled in the spleen and blood of the same mice. This is likely due to the high diversity of antibody sequences in both datasets. Interestingly, while single cell sequencing overlap with bulk sequencing is relatively larger in the spleen, the larger diversity of the blood repertoire implies a much smaller overlap between the two techniques.

We also explored structural predictions using AlphaFold3 (56). Overall, while our results demonstrate that high ipTM values can serve as a good indicator of binding when present, the low ipTM values observed for many true binders reflect a broader challenge in using AlphaFold3 for precise binding predictions. Given the growing interest in computational methods for antibody discovery (61), these findings suggest that AlphaFold3 could be a useful tool for pre-screening, but further refinements are required to improve its predictive accuracy in this context.

In conclusion, this study provides a framework for the computational detection of antigen-specific antibodies, validated through experimental methods. The ability to identify promising binders from bulk repertoire data, combined with insights into convergent selection, offers new opportunities for accelerating the discovery of therapeutic antibodies. With ongoing improvements, this pipeline could play a critical role in advancing the field of antibody therapeutics, not only for GFRAL but for a wide range of clinically relevant targets.

## 4 Materials and methods

### 4.1 Antigen preparation

The extracellular domain of human GFRAL(residues 19-351; NP_997293.2) was fused to the diphtheria toxin catalytic domain (DTA) with a C-terminal His-tag. The sequence was codon-optimized for expression in human cells and synthesized by GeneArt. The construct was cloned into a mammalian expression vector containing a CMV enhancer/promoter and SV40 polyA signal. Protein production and purification were performed by EVOTEC. Briefly, the protein was expressed in HEK293 cells and purified from clarified supernatant using metal affinity chromatography followed by Size Exclusion Chromatography (SEC). The final product was formulated in DPBS 1x buffer and sterile filtered. Protein concentration was determined by UV spectroscopy, and analytical SEC showed 96.7% purity. Reduced capillary electrophoresis revealed glycosylation-related heterogeneity. Sterility and endotoxin level lower than 1 EU/mg were validated. Finally, peptide mapping confirmed protein identity.

### 4.2 Mice and immunization

Trianni mice are C57BL/6-derived transgenic mice engineered to express human VH and V*κ* variable domains in place of the corresponding mouse loci. Five Trianni mice were purchased from Taconic Laboratory for experimentation at 6 to 8 weeks of age. During the study, animals were housed 5 per cage, the room was illuminated by lights set to give a 12 h light-dark cycle (on 06.00, off 18.00). The room was air-conditioned by a system designed to maintain an air temperature range of 22 ± 2*C* and a relative humidity range of 55 ± 15%. Diet and water were given *ad libitum*. Mouse experiments were conducted in an AAALAC-accredited facility in full compliance with the European Directive 2010/63/EU, transposed laws (Decree 2013/118), and the three Rs recommendations for the use of animals in biomedical research. All experimental protocols were approved by the French Ethics Committee for the Protection of Laboratory Animals (CEEA-022) and authorized by the French Ministry of Research (reference #59003). Female Trianni mice (5 per group) received intraperitoneal injections of 200 *μ*L per mouse of a 1:1 mixture of hGFRAL(19-351)::DTA-HIS antigen and Sigma Adjuvant System (S.A.S, Sigma S6322), prepared according to the manufacturer’s recommendations. Each injection contained 100 *μ*g of antigen, corresponding to a final antigen concentration of 0.5 mg/mL in the injected emulsion. Booster injections with identical formulations were administered on days 21, 42, and 63. All injections were delivered in a total volume of 200 *μ*L. The DTA (Diphtheria Toxin A) tag was incorporated to enhance antigen presentation and stimulate a more robust immune response. Blood draws were performed from the submandibular (facial) vein at days -3, 17, 25, 38, 46, 60, and 67 post-initial immunization for further analysis. Four days after the terminal injection, mice were euthanized. The animals were placed under deep general anesthesia by inhalation of 4% isoflurane. Once anesthesia was confirmed by the loss of reflexes (pupillary reflex and pinching withdrawal reflex), exsanguination was performed via transthoracic intracardiac puncture, which simultaneously allowed for the collection of terminal blood samples. A cervical dislocation was then performed to confirm death. The animal’s death was systematically confirmed by determining that irreversible cardiorespiratory arrest had occurred. Sera were collected by intracardiac puncture. Spleens were harvested in Phosphate Buffered Saline 1% bovine serum albumin for flow cytometry.

### 4.3 Bulk and single cell repertoire sequencing

Blood samples collected at different time points (day -3, day 17, day 39, day 60 and day 67) were processed for bulk B cell receptor (BCR) sequencing. We performed bulk sequencing on both heavy and light chain, using high-throughput sequencing technologies. Cells were FACS-sorted directly in 700 *μ*l buffer *RLT* + 1%*β* − *OH* and RNA extraction was performed in bulk using the RNeasy Micro kit (*QIAGEN*, #74034). Reverse transcription was made using oligos specifically targeting the constant regions of IgGs (IgG1, IgG2a, IgG2b, IgG2c and IgG3) and IgK mRNA molecules, and cDNAs were amplified by two steps of PCRs with custom oligos. PCR products were sent to a CRO (Eurofins Genomics) for indexing and sequenced using the Illumina NovaSeq 6000 (2*x*250*bp*) platform.

Single cell RNA sequencing (scRNA-seq) was performed using the 10x Genomics system to capture paired heavy and light chain information. Single B cells (Sbc) from blood samples (day 25, day 46 and day 67) as well as Sbc from the spleens were processed following the 10x Genomics protocol for single cell V(D)J sequencing (Chromium Next GEM Single Cell 5’ Reagent Kits v2 Dual Index). Briefly, cells were FACS-sorted in a 96-well plate and centrifuged 5 minutes at 400xg. After removing the supernatant, cells were resuspended with 36.8 *μ*l PBS and mixed with 36.3 *μ*l RT master mix before loading into the chip K for the encapsulation using the chromium controller. Libraries were prepared using the 10x Genomics Single Cell V(D)J Kit, and sequencing was performed on an Illumina NovaSeq 6000 (2x100 bp) platform. This allowed for the generation of paired heavy and light chain data, which were used to match the heavy chains identified from the bulk sequencing.

The antigen-positive and antigen-negative populations were obtained with the following procedure: after the mouse was sacrificed, splenic B cells were enriched from splenocytes by magnetic negative selection using Pan B Cell Isolation Kit II (Miltenyi; 130-101-638) with addition of anti-IgM Microbeads according to manufacturers’ instructions. After enrichment, cells were suspended in a buffer solution and stained with 1 μg per 10^8^ cells of the following antibodies: Rat anti-mouse IgG1 brilliant violet 421, Rat anti-mouse IgG2a/b brilliant violet 510, rat anti-mouse CD45R brilliant violet 786, rat anti-mouse CD138 and rat anti-mouse IgD, rat anti-mouse IgM, rat anti-mouse CD4, rat anti-mouse CD8 and rat anti-mouse F4/80 brilliant violet 605 (dump channel). For antigen-specific sorting, cynoGFRAL was detected using anti-His-AF647. Cells were separated using BD FACSAria Fusion (BD Biosciences) for flow cytometric cell sorting to isolate IgG antigen-specific B cells or non-specific B cells. Cells were distributed into 10X chips and processed on the 10X Genomics instrument.

### 4.4 IgG preparation and biophysical characterization

IgG supernatants were generated using Linear Expression Cassettes (LEC) as previously described (62). Briefly, antibody heavy and light variable region gene fragments were synthesized (Twist Bioscience) with specific overhangs: 5’ sequences overlapping the 3’ end of a CMV promoter, and 3’ sequences complementary to the 5’ end of respective constant region gene fragments. LECs were assembled by combining three separate gene fragments (promoter with 5’UTR, variable domain, constant domain with 3’ UTR) through overlap-extension PCR. Expi293F™ cells were cultured in Expi293 expression medium (ThermoFisher, A1435101) and were transfected at final density of 3 × 10^6^ cells/ml with heavy and light chain LECs using ExpiFectamine 293 transfection kit (Gibco). ExpiFectamine™ 293 enhancers were added 20h after transfection. After 5 days, supernatants were collected, and antibody concentrations were determined by Bio-layer interferometry before concentration adjustment to 20 *μ*g/ml.

Heavy and light chain DNA sequences were synthesized as string DNA fragments and cloned into eukaryotic expression vectors in frame with human heavy or light chain constant domains (GENEWIZ, Azenta Life Sciences). Expi293F™ cells (75 millions) were co-transfected with 15 *μ*g of each plasmid encoding the respective IgG chains using ExpiFectamine™. ExpiFectamine™ 293 enhancers were added 20 h after transfection. Culture media were harvested after 6 days, filtered, and purified using MabSelect SuRe™ columns (Repligen). Following elution, IgGs were desalted and formulated in His buffer at 1 mg/ml.

#### 4.4.1 Characterization

Each batch underwent comprehensive analysis via multiple chromatographic methods: size exclusion chromatography (Superdex 200 5/150 GL columns, GE Healthcare), hydrophobic interaction chromatography (MabPac HIC-10 5 μm column, ThermoFisher), and cation exchange chromatography (Tosoh TSKgel SP-STAT column) using a 1290 Infinity II Bio LC System (Agilent). Additionally, capillary electrophoresis-sodium dodecyl sulfate (cE-SDS) analysis was performed under both reducing and non-reducing conditions on a LabChip GX caliper.

### 4.5 Perplexity estimation

We estimate the humanness of antibody candidates using the perplexity score, calculated using the IgLM model (63). IgLM is an autoregressive generative model that was trained on 558 million human antibody heavy and light chain sequences from the Observed Antibody Space OAS database (64). Given an amino acid sequence *x* of length *L*, we denote as $x_{<i}=x_{1},\ldots x_{i-1}$ the partial sequence corresponding to the prefix of length *i* − 1 of sequence *x*. IgLM is trained to estimate $p\left(x_{i}|x_{<i}\right)$. The log probability logp(x) that IgLM generates a sequence *x* is computed as:

(1) $$\log p\left(x\right)=\sum_{i=1}^{L}\log p\left(x_{i}|x_{i-1}\right).$$

From the log likelihood in Equation 1, the perplexity score is computed as:

(2) $$\text{Perplexity}\left(x\right)=\exp\left[-\frac{1}{L}\log p\left(x\right)\right],$$

where we divide scores by the length of the sequence to allow comparisons between sequences of different lengths. The lower the perplexity in Equation 2, the more human-like the antibody sequence appears to IgLM. To compute perplexity, we used the function provided directly by the IgLM package. The perplexity of the heavy and light chains are computed independently and multiplied together.

### 4.6 Solubility score estimation

To compute an estimate of the solubility of the antibodies, we use the model from (37, 65). The solubility of each chain is modeled as:

(3) $$f_{\text{sol}}=-\sum_{i=1}^{L}\text{SASA}\left(i,x_{i}\right)\text{HW}\left(x_{i}\right)+\text{const},$$

where SASA(*i*, *x_i_*) is the solvent accessible surface area of the residue of type *x_i_* at position *i*, and HW(*x_i_*) is the hydrophobic weight of a residue of type *x_i_*.* A* convolutional neural network was trained on a dataset containing antibodies from the SabDab database (66) to predict the per-residue SASA given the heavy and light chain sequences of the antibody. From the per-residue SASA, we compute the solubility score for each chain given Equation 3, and sum the scores of the two chains to get a score for the antibody.

### 4.7 Surface plasmon resonance binding

Surface Plasmon Resonance (SPR) binding experiments were conducted on the Carterra LSA platform using a capture setup in 1× HBS-EP+ buffer (10 mM HEPES, pH 7.4, 150 mM NaCl, 3 mM EDTA, 0.05% v/v Surfactant P20; GE Healthcare, BR100669) at 25°C. For chip preparation, goat anti-human Fc antibody (SouthernBiotech, 2048-01) was immobilized onto a CMDP chip (Carterra) by amine coupling. The goat anti-human Fc antibody was prepared in 10 mM acetate (pH 4.5) at 75 *μ*g/ml, resulting in 2000 response units (RU). Crude antibody extracts were captured on the sensor chip at 0.5 *μ*g/ml for 15 minutes. To assess antibody binding against GFRAL, a concentration series of GFRAL (100 pM to 500 nM) was flowed over each flow cell for 5 minutes. GFRAL binding was monitored for 5 minutes, followed by a 30-minute dissociation phase. All samples were diluted in HBS-EP+ running buffer. The surface was regenerated with two 40-second pulses of 10 mM glycine-HCl (pH 2). Blank reference sensorgrams were obtained using buffer without GFRAL. All sensorgrams were double-referenced by subtracting both blank injection and reference surface signals. Binders were defined based on a %*R*_max_ higher than 30, non-binders as having %*R*_max_ less than 10. The concentration series sensorgrams were fitted using a 1:1 Langmuir model with Kinetics software (Carterra).

## Data Availability

The data has been released. https://doi.org/10.5281/zenodo.22711596. The raw SPR data is available in the SI Dataset 1. The list of validated GFRAL-antibody sequences is available in the SI Dataset 2.

## Supplementary material

Supplementary Figure 1 Sequence similarity of the 40 STAR hits found in the single cell data. A network of similarity betweenthe 40 STAR hits found in the single cell data, including clusters formed by one antibody, confirms that the STAR hits chosento be tested by SPR are dissimilar in sequence to each other. They are, by definition of how STAR hits are called, similar toother bulk sequences that were not used when making this plot. Each point in the network is a VH and VL sequence sequence,colored by binding (orange) or non-binding (purple) status as identified by SPR. Clones (VH+VL) are in same VH/VL clusterif they have the same closest Heavy V germline Group, same closest Light V germline Group, same heavy CDR3 length andsame light CDR3 length. Since the purpose of the plot is to asses sequence similarity of the STAR hit binders, for visualizationpurposes we focus on binders only a few examples of non-binders are shown, whereas 12 non-binders that do not cluster withthe binders are not shown.

Supplementary Figure 2 *k*_off_ as a function of *K_D_* colored by the number of mice and organs they were found in. Scatter plot showing the relationship between *k*_off_ and *K_D_* of the SPR validated sequences. Sequences observed in single mice are colored in red (mouse 1), blue (mouse2) and green (mouse 3), with the same hues as in **Figure 4A** of the main text for different tissue sources (blood or spleen) and days. Sequences shared by two mice are shown in orange, and three mice in yellow.

Supplementary Figure 3 Lineage trees. Lineage trees for of the different mice reconstructed with the sorted positive (squares) and negative (circles) sequences. The data come from splenocyte single cell data sorted into antigen-positive and antigen-negative cells andthe lineages are chosen as those that are shared by all three mice. Each lineage was colored based on the CDR3 amino acidsequence, with shades representing sequence variants. Branch lengths are proportional to the number of mutations. Greydiamonds denote the distinct germline ancestor of each tree. Node sizes represent the count of sequences within the lineage inthe splenocytes single cell sorted data. Nodes with asterisks are STAR hits.

Supplementary Figure 4 Fraction of positive in the lineage. Scatter plot showing the relationship between lineage size and the fractionof antigen-specific sequences within each lineage. Each point represents a lineage, with the x-axis indicating the number of sequences in the lineage (on a linear scale) and the y-axis representing the fraction of those sequences that were identified as antigen-specific. The data is color-coded by mouse. A general trend is observed where larger lineages tend to contain a higher fraction of antigen-specific sequences, suggesting a potential enrichment of antigen-specific B cells in expanded clonal families.

Supplementary Figure 5 (see next pages) Kinetic binding analysis and affinity profiling of 246 GFRAL antibody clones. **(A)** Binding hit rate assessment. Of 246 clones screened, 72 clones (29%) demonstrated binding to the target antigen. **(B)** Affinity distribution of binders. Kinetic analysis revealed that 57 of 72 binding clones (79%) exhibited dissociation constants (*K_D_*) in the 0.1–100 nM range, indicating high to moderate binding affinity. **(C)** Representative binding kinetics. Surfaceplasmon resonance (SPR) sensorgrams illustrating the range of association (*k_a_*) and dissociation (*k_d_*) rates observed across different affinity categories. Sensorgrams display analyte injection followed by dissociation phases, with fitted curves for kineticparameter determination.

Supplementary Figure 6 (see next pages) Biophysical characterization of the top 10 GFRAL antibodies. **(A)** Sample yield of GFRAL antibodies compared to Trastuzumab control. **(B)** Comprehensive quality assessment of the 10 purified GFRAL antibodiesusing Trastuzumab as reference standard. Orthogonal analytical methods evaluated antibody quality attributes: size exclusionchromatography (SEC) for detection of fragments, multimers, and aggregates; hydrophobic interaction chromatography (HIC) for assessing hydrophobic heterogeneity; analytical cation exchange chromatography (aCEX) for evaluation of charge variants;and capillary electrophoresis sodium dodecyl sulfate (cE-SDS) under reducing and non-reducing conditions for analysis ofstructural integrity. The 95% pass criterion is indicated. **(C)** Representative analytical profiles showing SEC, HIC and aCEX chromatograms and cE-SDS under reducing and non-reducing conditions for Trastuzumab and two GFRAL antibody clones:CL-1440303 (lowest quality profile) and CL-1440286 (optimal quality profile).

Supplementary Table 1 Overview of the number of reads/cells and unique CDR3 sequences for each mouse, tissue, day, and sequencingtype.
