# Supplementary material for: Integrating computational and experimental approaches to identify antigen–specific antibodies
Source: Front Immunol. 2026 Oct 29;17:1951538. doi: 10.3389/fimmu.2026.1951538 (PMC13755276; doi:10.3389/fimmu.2026.1951538)

Integrating computational and experimental approaches to identify antigen–specific antibodies.
Supplementary information

Maria Francesca Abbate,[1] Paul Pereira,[1,2] Pierre Toxe,[2] Nicolas Maestrali,[2] Marie Gagnaire,[2] Emmanuelle Vigne,[2] Melody A. Shahsavarian,[2] Thierry Mora,[1,*] and Aleksandra M. Walczak[1,*]

[1] *Laboratoire de physique de l'École normale supérieure, CNRS, PSL University, Sorbonne Université, and Université de Paris, 75005 Paris, France*

[2] *Large Molecule Research, Sanofi, Vitry-sur-Seine, France*

* Corresponding authors. These authors contributed equally.

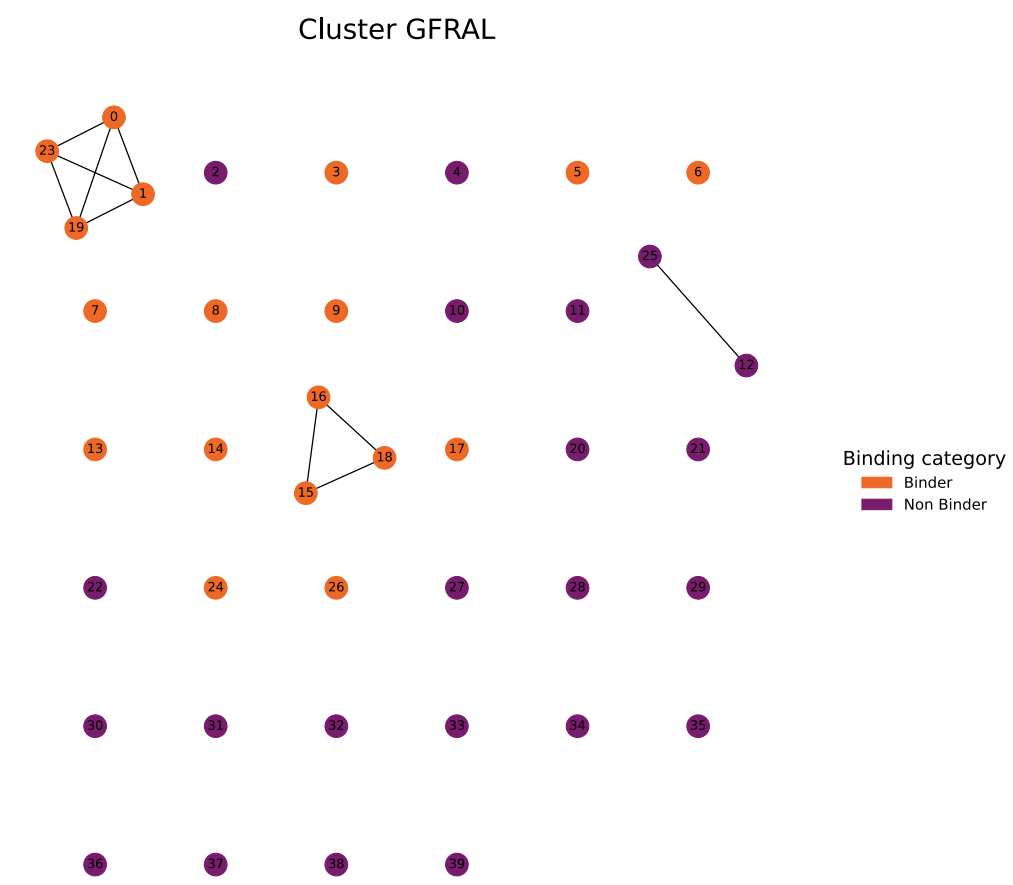

FIG. S1. **Sequence similarity of the 40 STAR hits found in the single cell data.** A network of similarity between the 40 STAR hits found in the single cell data, including clusters formed by one antibody, confirms that the STAR hits chosen to be tested by SPR are dissimilar in sequence to each other. They are, by definition of how STAR hits are called, similar to other bulk sequences that were not used when making this plot. Each point in the network is a VH and VL sequence sequence, colored by binding (orange) or non-binding (purple) status as identified by SPR. Clones (VH+VL) are in same VH/VL cluster if they have the same closest Heavy V germline Group, same closest Light V germline Group, same heavy CDR3 length and same light CDR3 length. Since the purpose of the plot is to asses sequence similarity of the STAR hit binders, for visualization purposes we focus on binders only a few examples of non-binders are shown, whereas 12 non-binders that do not cluster with the binders are not shown.

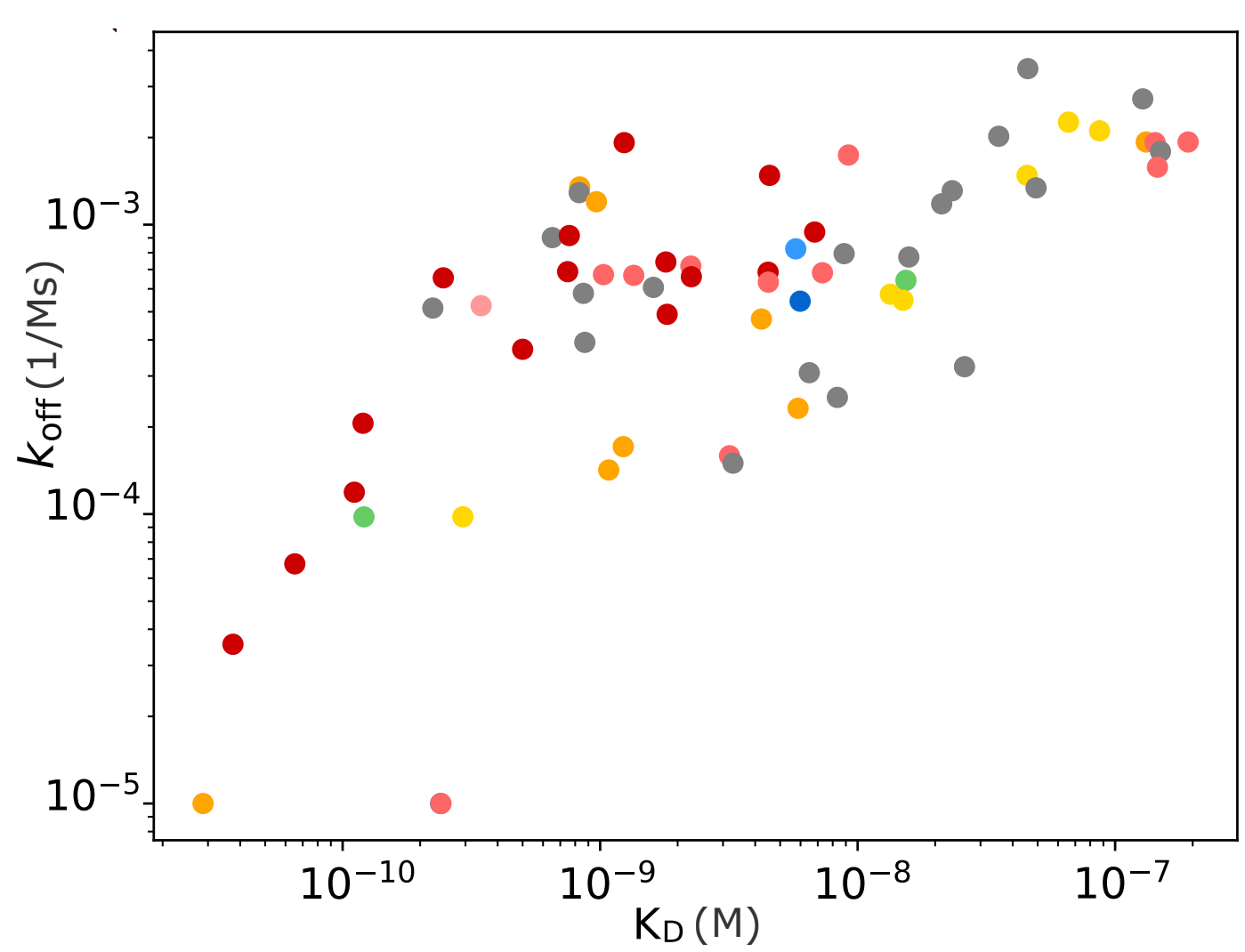

FIG. S2. k_{off} **as a function of** K_D **colored by the number of mice and organs they were found in.** Scatter plot showing the relationship between k_{off} and K_D of the SPR validated sequences. Sequences observed in single mice are colored in red (mouse 1), blue (mouse2) and green (mouse 3), with the same hues as in Fig. 4A of the main text for different tissue sources (blood or spleen) and days. Sequences shared by two mice are shown in orange, and three mice in yellow.

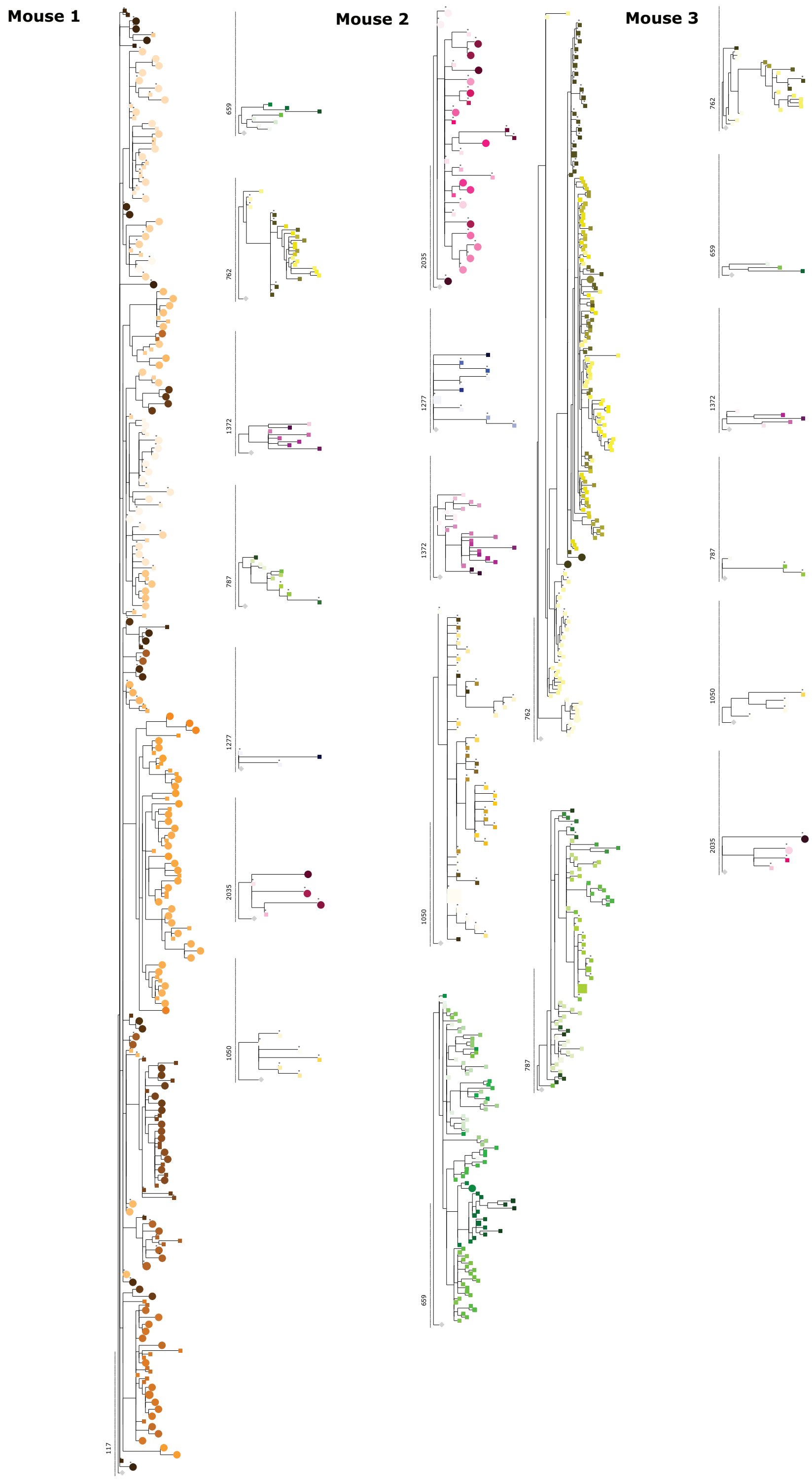

FIG. S3. **Lineage trees.** Lineage trees for of the different mice reconstructed with the sorted positive (squares) and negative (circles) sequences. The data come from splenocyte single cell data sorted into antigen-positive and antigen-negative cells and the lineages are chosen as those that are shared by all three mice. Each lineage was colored based on the CDR3 amino acid sequence, with shades representing sequence variants. Branch lengths are proportional to the number of mutations. Grey diamonds denote the distinct germline ancestor of each tree. Node sizes represent the count of sequences within the lineage in the splenocytes single cell sorted data. Nodes with asterisks are STAR hits.

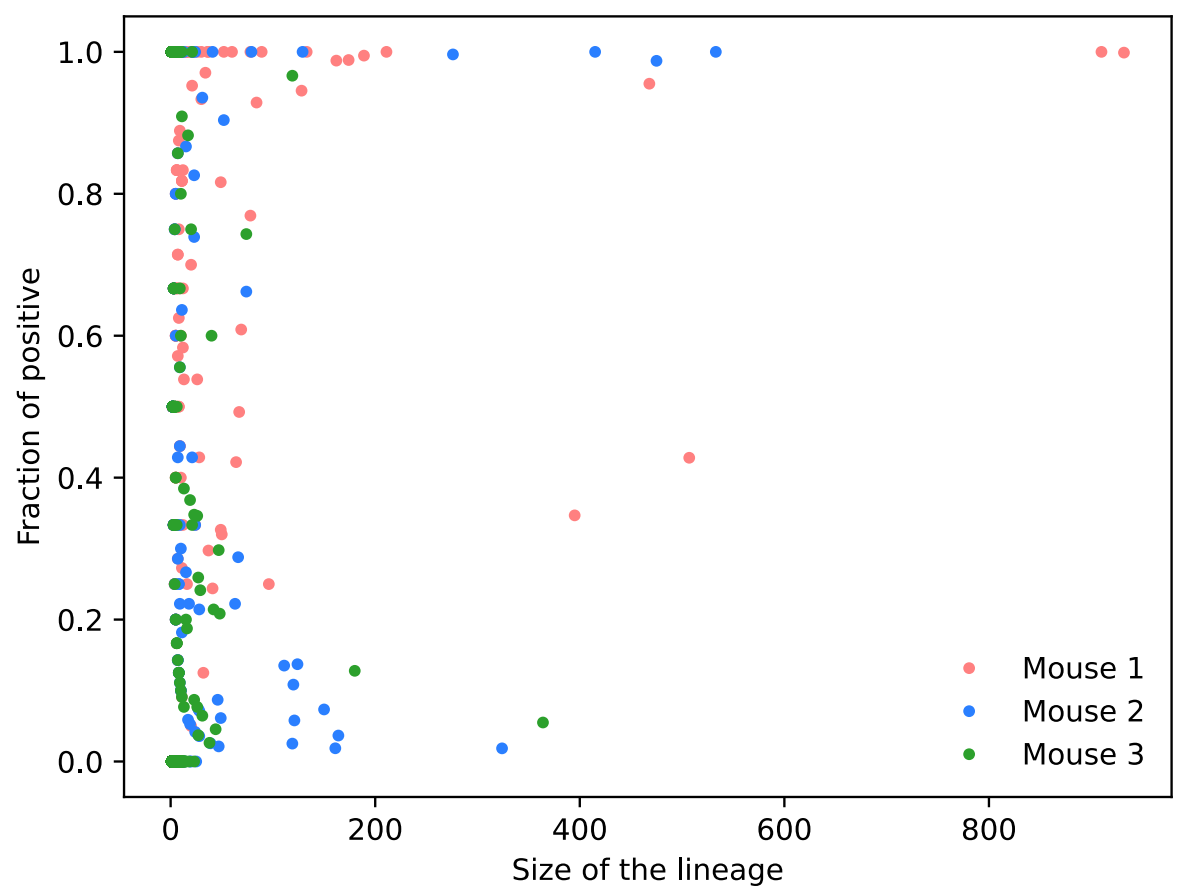

FIG. S4. **Fraction of positive in the lineage.** Scatter plot showing the relationship between lineage size and the fraction of antigen-specific sequences within each lineage. Each point represents a lineage, with the x-axis indicating the number of sequences in the lineage (on a linear scale) and the y-axis representing the fraction of those sequences that were identified as antigen-specific. The data is color-coded by mouse. A general trend is observed where larger lineages tend to contain a higher fraction of antigen-specific sequences, suggesting a potential enrichment of antigen-specific B cells in expanded clonal families.

FIG. S5. (see next pages) **Kinetic binding analysis and affinity profiling of 246 GFRAL antibody clones.** **(A) Binding hit rate assessment.** Of 246 clones screened, 72 clones (29%) demonstrated binding to the target antigen. **(B) Affinity distribution of binders.** Kinetic analysis revealed that 57 of 72 binding clones (79%) exhibited dissociation constants (K_D) in the 0.1-100 nM range, indicating high to moderate binding affinity. **(C) Representative binding kinetics.** Surface plasmon resonance (SPR) sensorgrams illustrating the range of association (k_a) and dissociation (k_d) rates observed across different affinity categories. Sensorgrams display analyte injection followed by dissociation phases, with fitted curves for kinetic parameter determination.

FIG. S6. (see next pages) **Biophysical characterization of the top 10 GFRAL antibodies.** **(A)** Sample yield of GFRAL antibodies compared to Trastuzumab control. **(B)** Comprehensive quality assessment of the 10 purified GFRAL antibodies using Trastuzumab as reference standard. Orthogonal analytical methods evaluated antibody quality attributes: size exclusion chromatography (SEC) for detection of fragments, multimers, and aggregates; hydrophobic interaction chromatography (HIC) for assessing hydrophobic heterogeneity; analytical cation exchange chromatography (aCEX) for evaluation of charge variants; and capillary electrophoresis sodium dodecyl sulfate (cE-SDS) under reducing and non-reducing conditions for analysis of structural integrity. The 95% pass criterion is indicated. **(C)** Representative analytical profiles showing SEC, HIC and aCEX chromatograms and cE-SDS under reducing and non-reducing conditions for Trastuzumab and two GFRAL antibody clones: CL-1440303 (lowest quality profile) and CL-1440286 (optimal quality profile).

MOUSE	DAY	TYPE	TISSUE	N° of Cell/Reads	N° of Unique CDR3
Mouse 1	D17	Bulk	Blood	3,903,140	94,453
Mouse 1	D25	Bulk	Blood	3,841,988	62,871
Mouse 1	D25	Single cell	Blood	1,358	1,289
Mouse 1	D39	Bulk	Blood	3,972,126	83,753
Mouse 1	D46	Bulk	Blood	3,878,641	68,974
Mouse 1	D46	Single cell	Blood	644	539
Mouse 1	D60	Bulk	Blood	1,276,181	26,926
Mouse 1	D67	Bulk	Spleen	3,675,017	73,662
Mouse 1	D67	Single cell	Blood	4,272	2,604
Mouse 1	D67	Single cell	Spleen	11,755	1,852
Mouse 2	D25	Bulk	Blood	4,161,213	68,690
Mouse 2	D25	Single cell	Blood	1,358	1,289
Mouse 2	D39	Bulk	Blood	3,575,899	73,227
Mouse 2	D46	Bulk	Blood	3,360,544	56,469
Mouse 2	D46	Single cell	Blood	651	565
Mouse 2	D60	Bulk	Blood	4,511,141	77,013
Mouse 2	D67	Bulk	Blood	3,850,030	71,053
Mouse 2	D67	Bulk	Spleen	3,767,658	70,434
Mouse 2	D67	Single cell	Blood	4,209	2,453
Mouse 2	D67	Single cell	Spleen	7,776	1,244
Mouse 3	D3	Bulk	Blood	3,313,716	60,136
Mouse 3	D17	Bulk	Blood	3,837,689	86,623
Mouse 3	D25	Bulk	Blood	4,420,976	81,634
Mouse 3	D25	Single cell	Blood	1,358	1,289
Mouse 3	D39	Bulk	Blood	3,390,867	67,828
Mouse 3	D46	Single cell	Blood	727	628
Mouse 3	D60	Bulk	Blood	3,827,782	51,971
Mouse 3	D67	Bulk	Spleen	3,938,011	70,673
Mouse 3	D67	Single cell	Blood	1,887	1,443
Mouse 3	D67	Single cell	Spleen	3,783	758

TABLE S1. Overview of the number of reads/cells and unique CDR3 sequences for each mouse, tissue, day, and sequencing type.

Figure S5 A-B

A. **Hit rate**

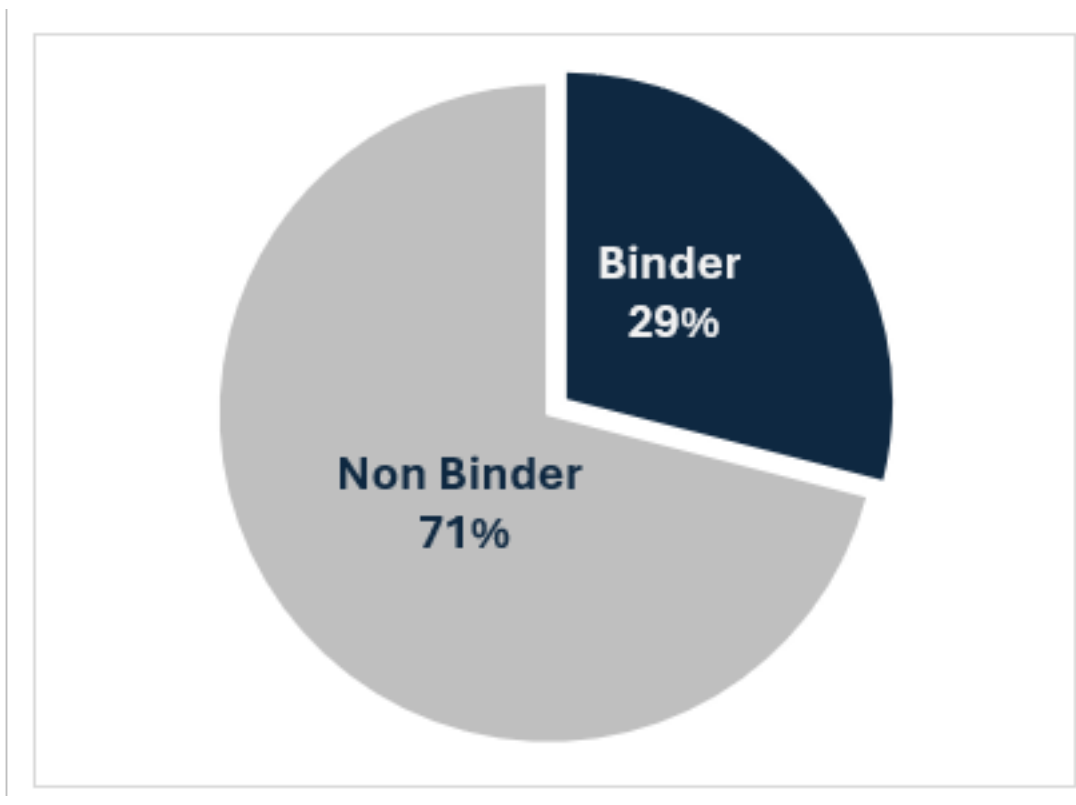

B. **Affinity distribution by clone origin**

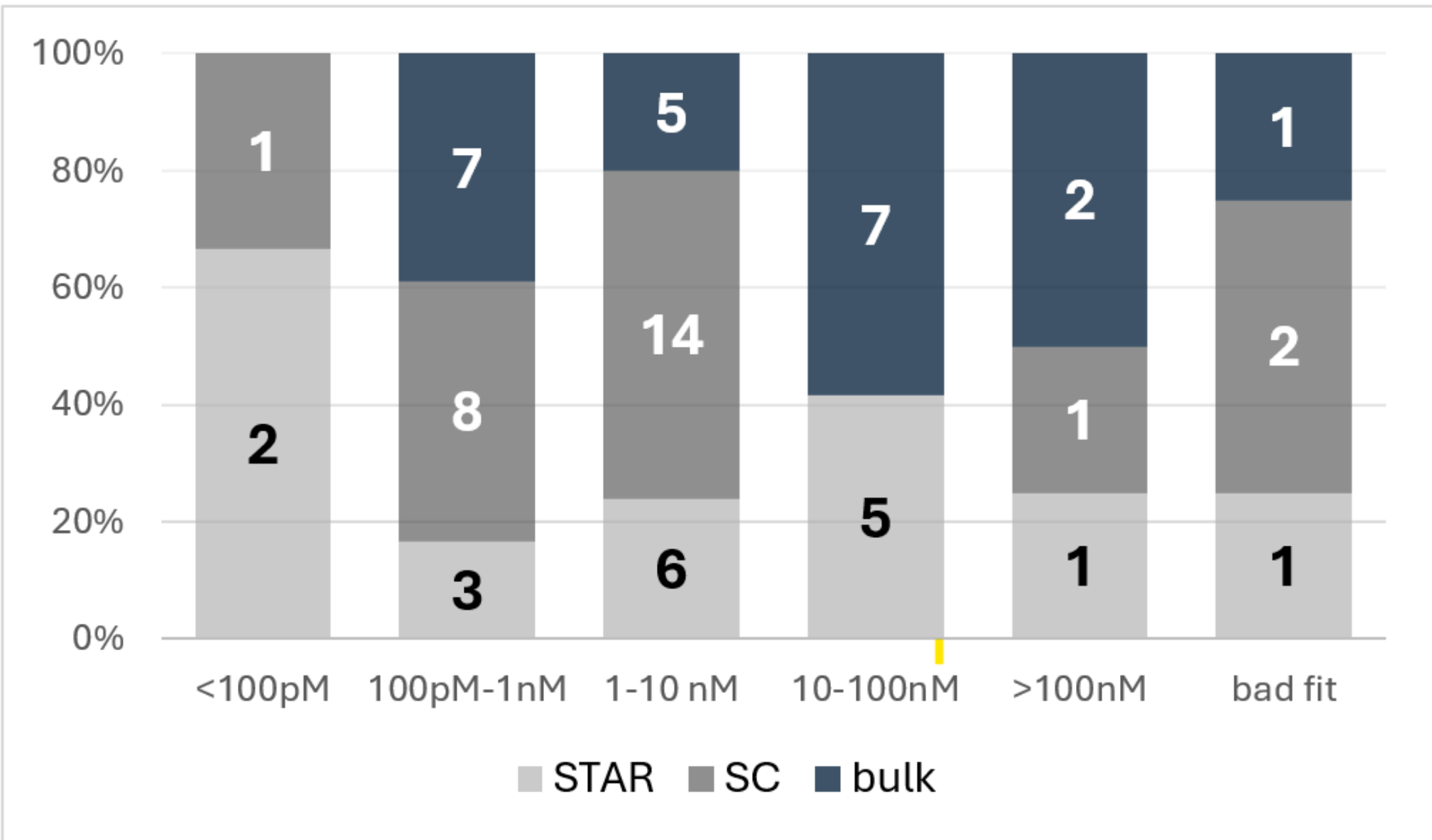

Figure S5 C

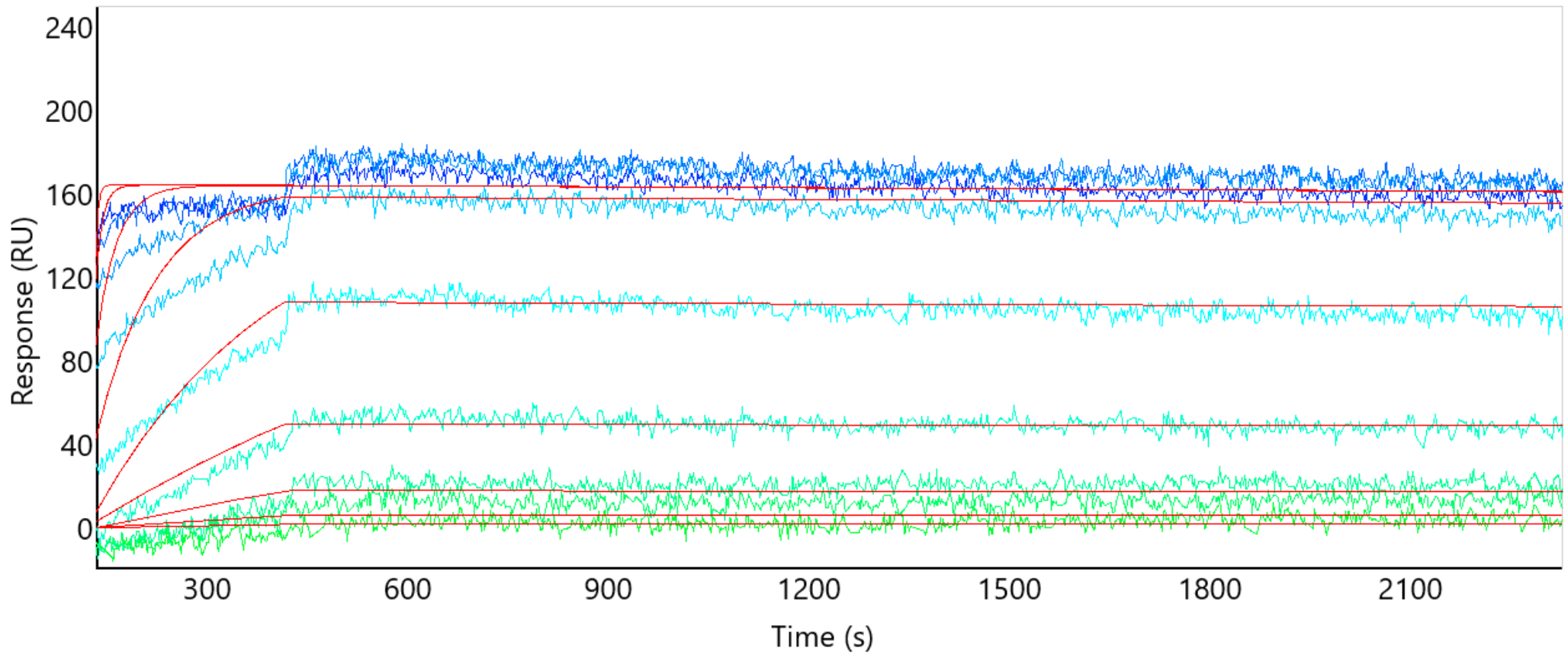

HIGH AFFINITY PROFILE

KD = 3 pM

slow kd = 1.00E-05 1/s

(approaching instrument limit)

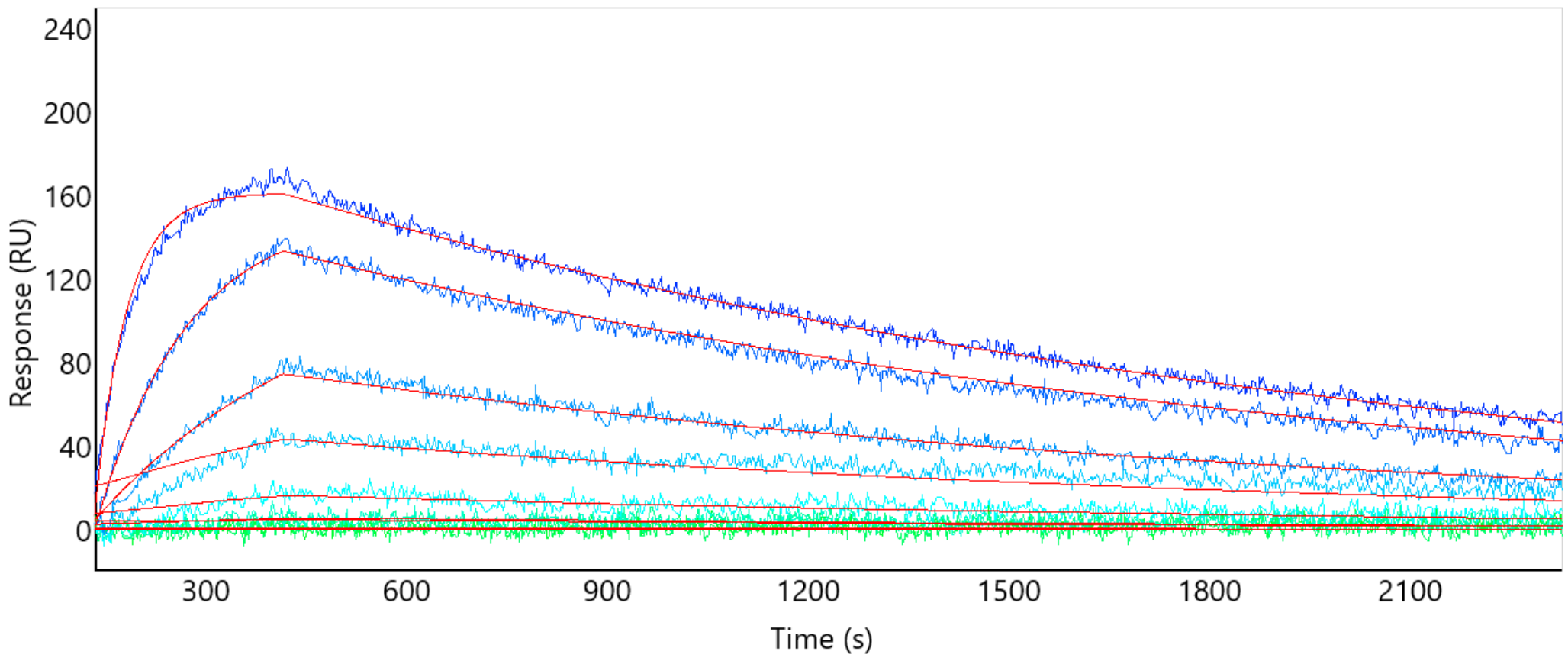

MEDIUM AFFINITY PROFILE

KD = 15 nM

kd = 6.42E-04 1/s

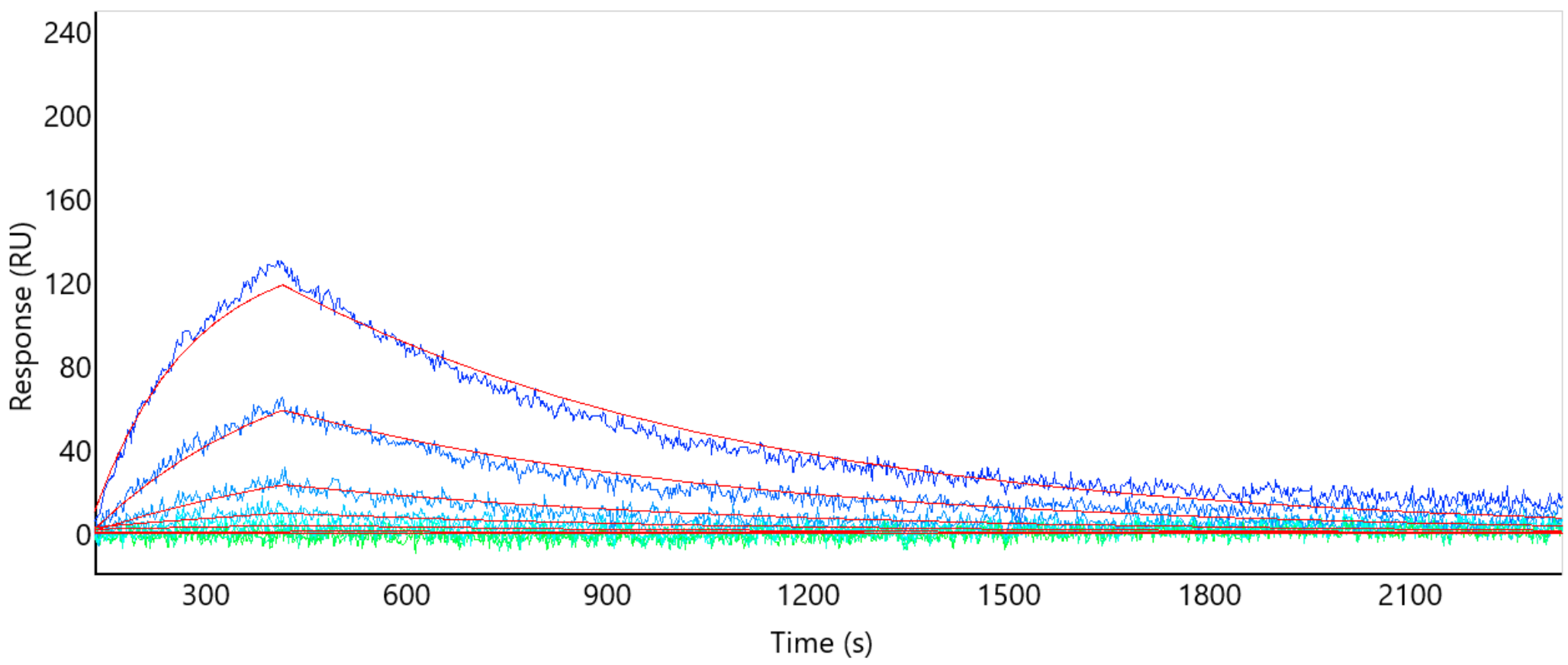

LOW AFFINITY PROFILE

KD = 132 nM

kd = 1.4E-03 1/s

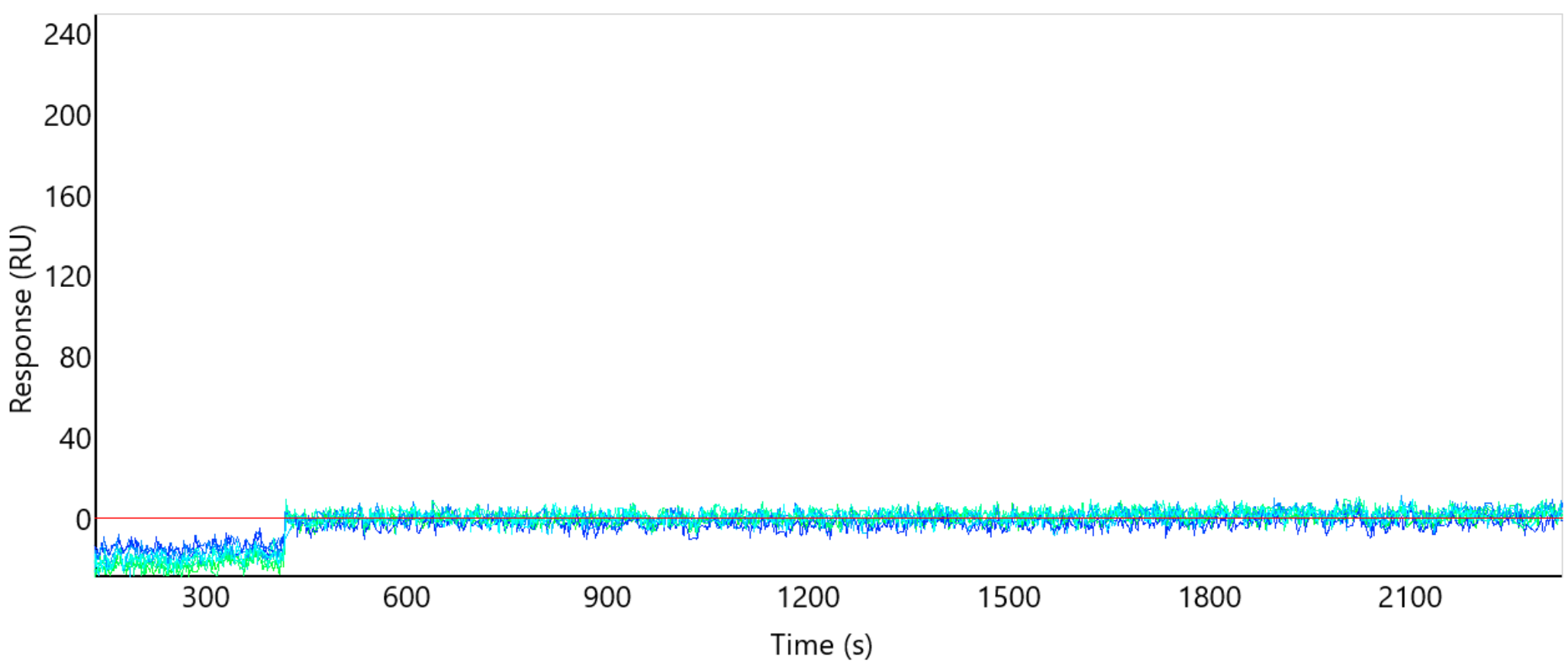

NON BINDER

Figure S6 A

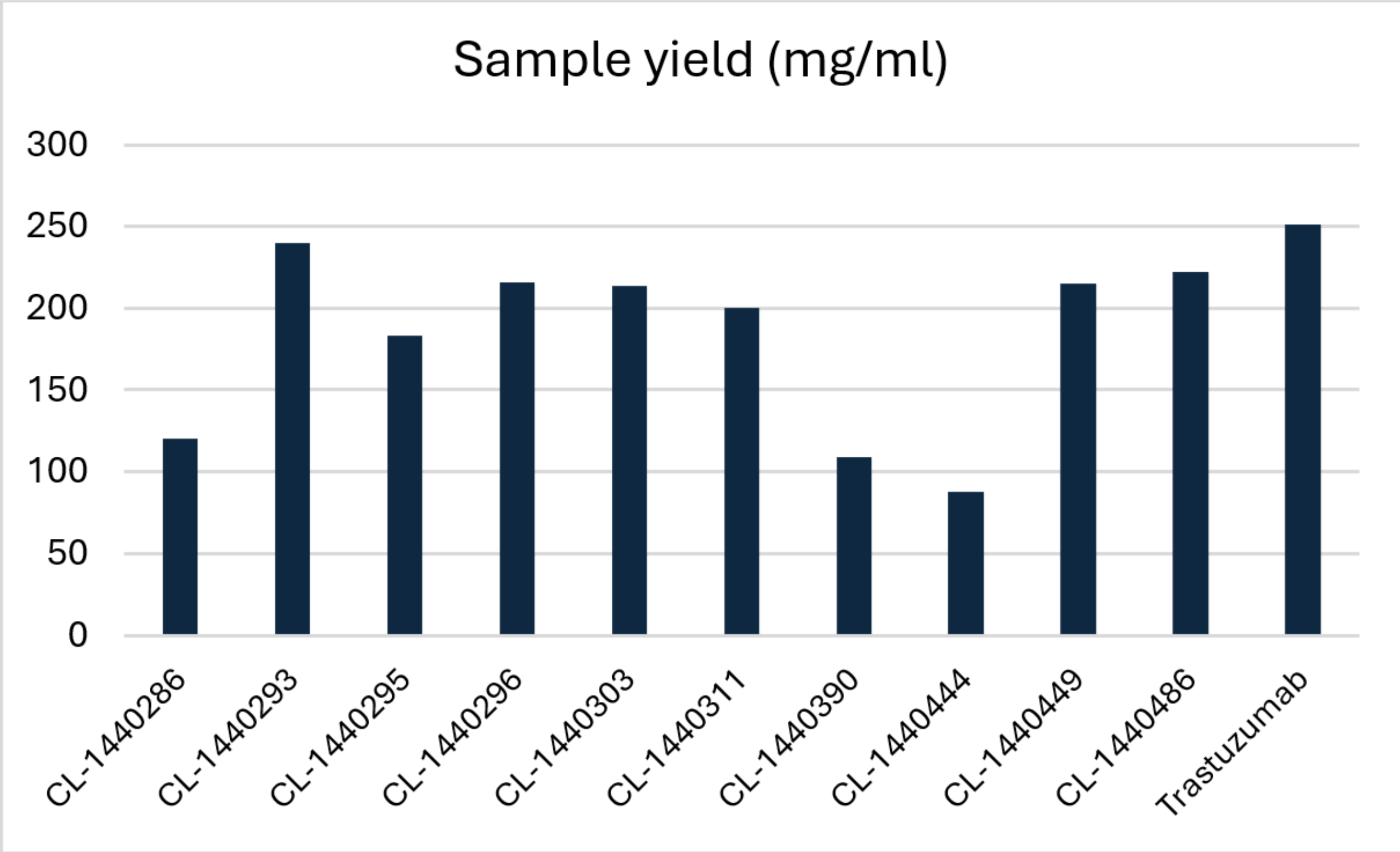

Figure S6 B

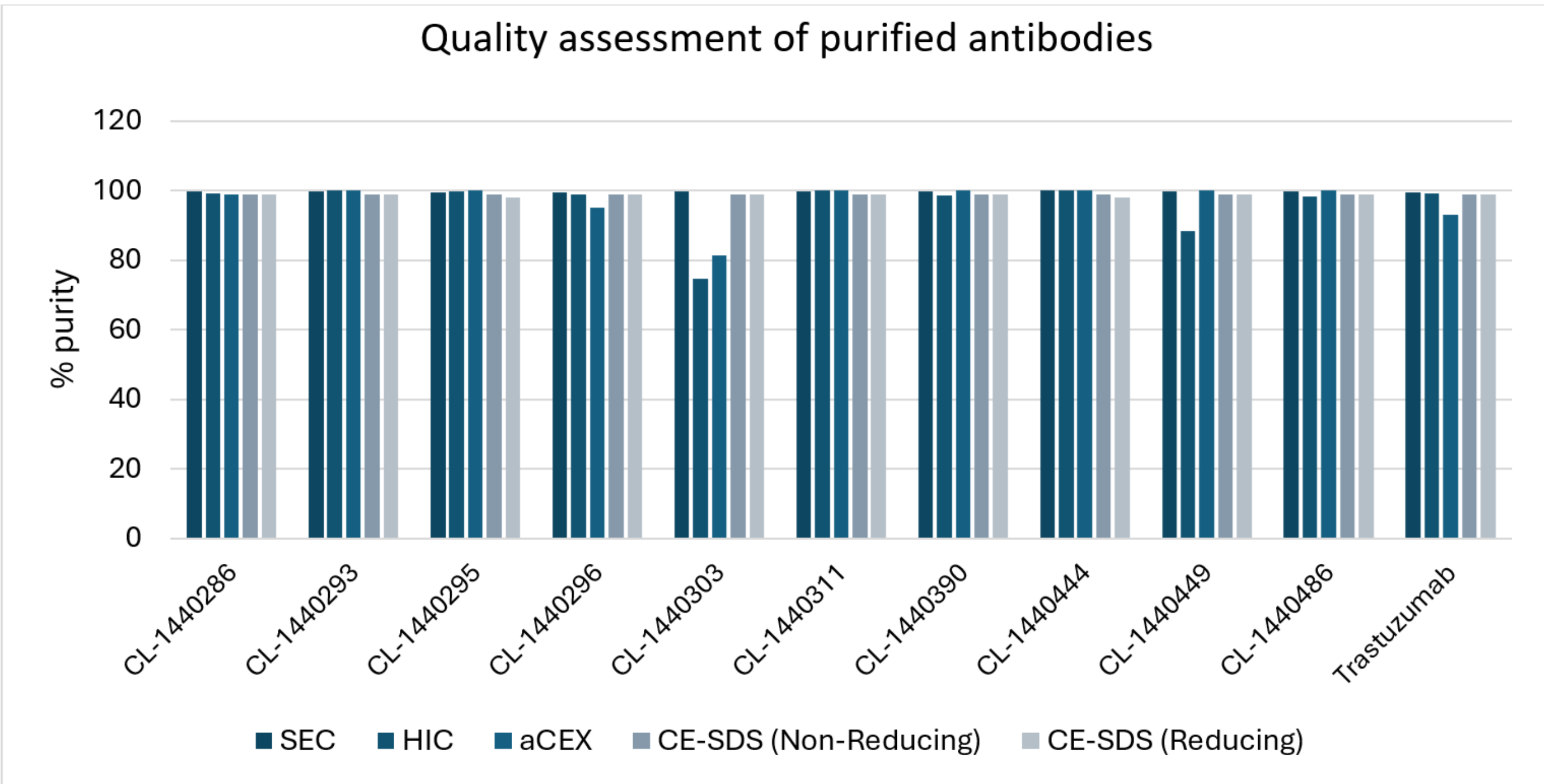

Figure S6 C - SEC

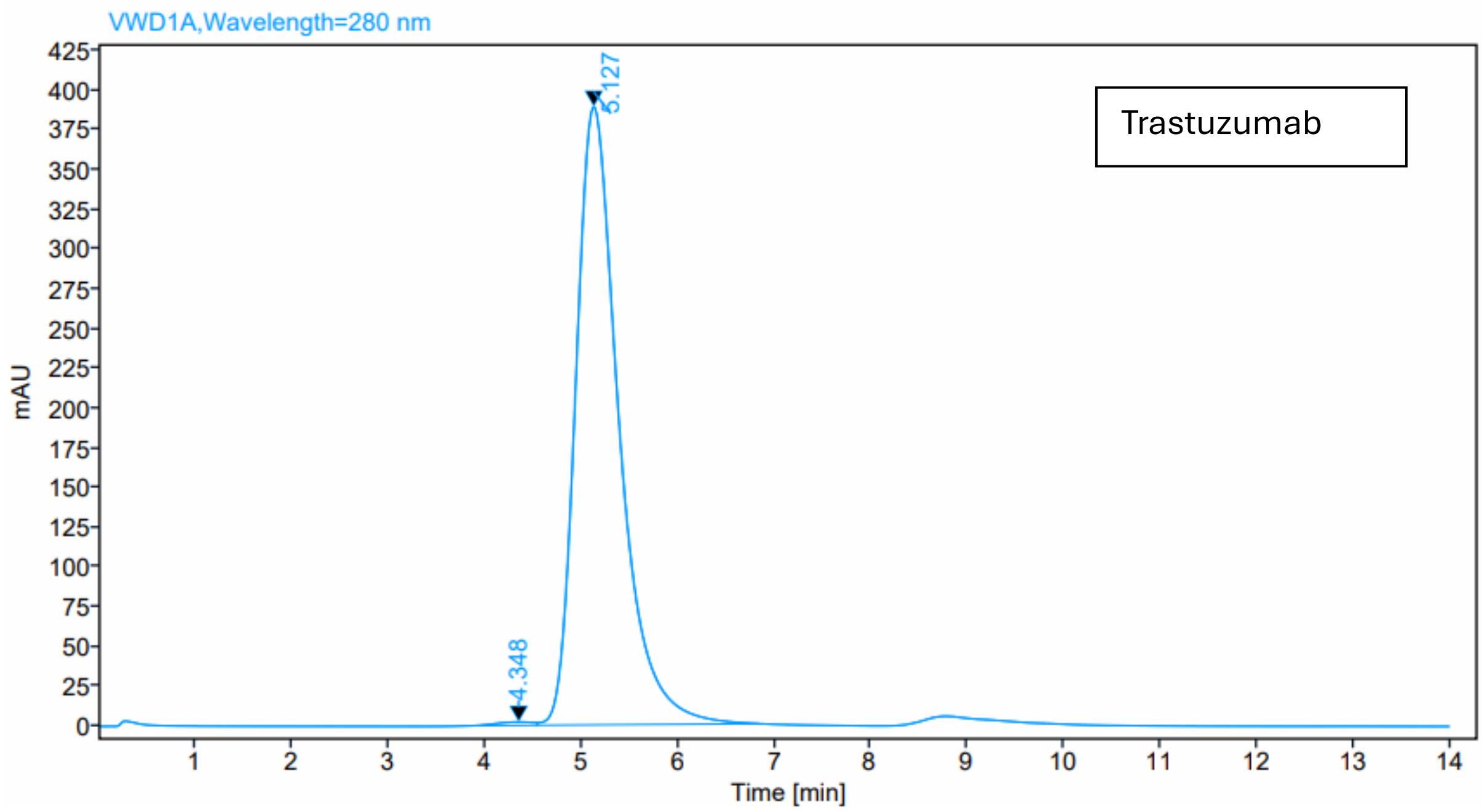

Signal: VWD1A,Wavelength=280 nm

Name	RT [min]	Area	Area%	Height	Width 50% [min]	Sample Dilution Factors
VA2-25-3865-1	4.35	55.2	0.5	2.1	0.44	1
VA2-25-3865-1	5.13	12112.4	99.5	389.0	0.46	1
	Sum	12167.5913				

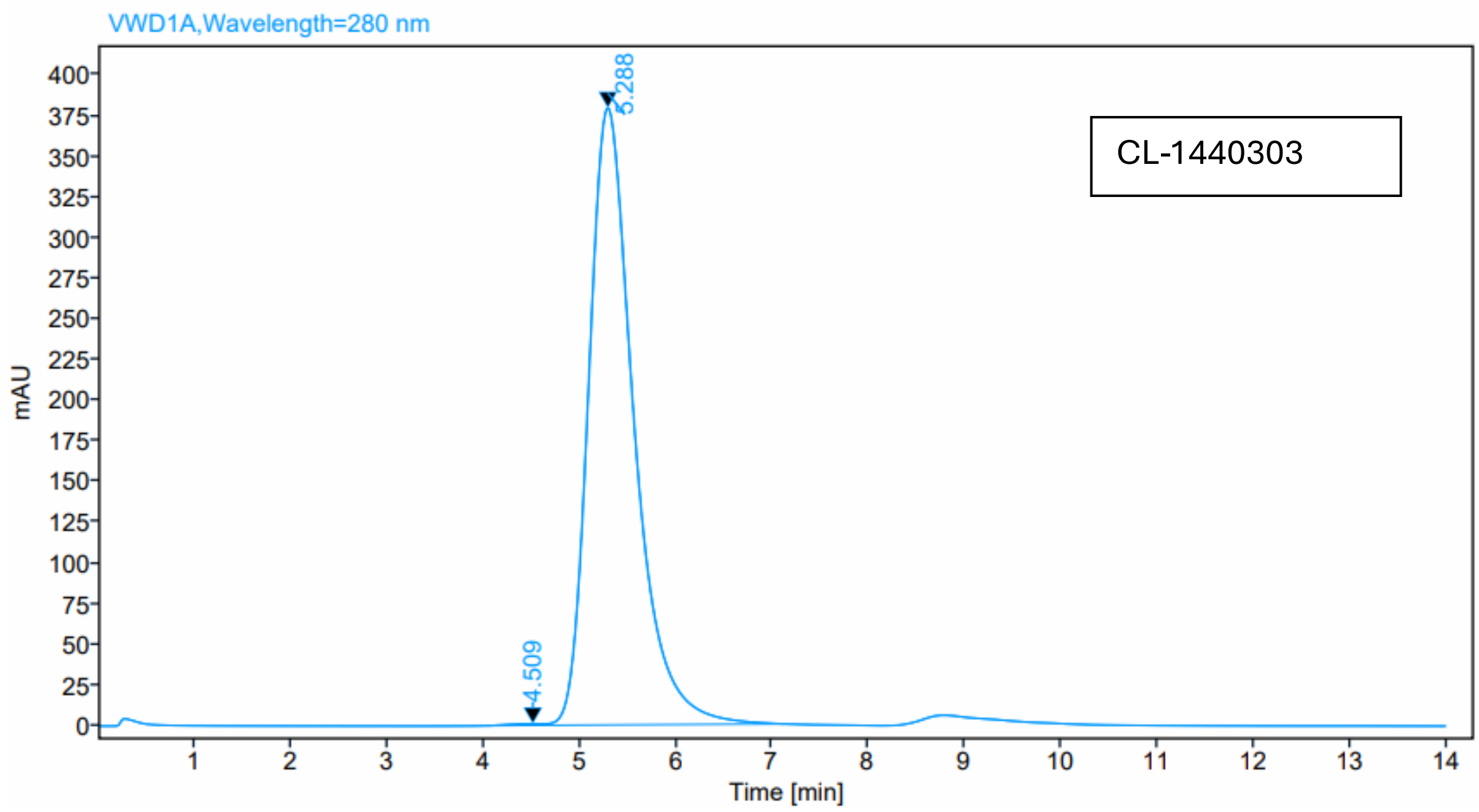

Signal: VWD1A,Wavelength=280 nm

Name	RT [min]	Area	Area%	Height	Width 50% [min]	Sample Dilution Factors
VA2-25-3859-1	4.51	23.2	0.2	1.0	0.35	1
VA2-25-3859-1	5.29	12416.9	99.8	379.2	0.48	1
	Sum	12440.0553				

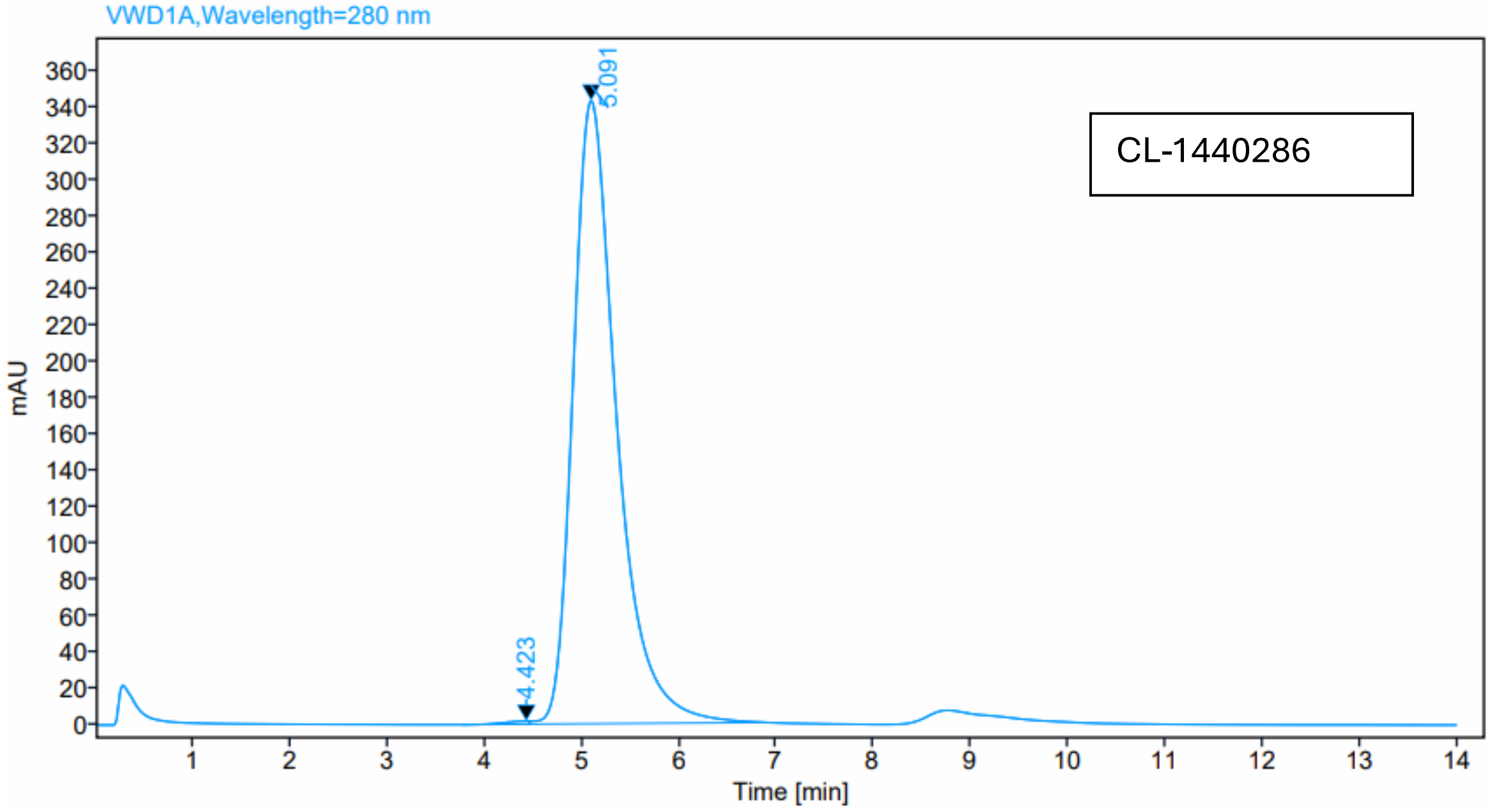

Signal: VWD1A,Wavelength=280 nm

Name	RT [min]	Area	Area%	Height	Width 50% [min]	Sample Dilution Factors
VA2-25-3855-1	4.42	29.1	0.3	1.7	0.26	1
VA2-25-3855-1	5.09	10729.9	99.7	343.3	0.46	1
	Sum	10759.0148				

Figure S6 C - HIC

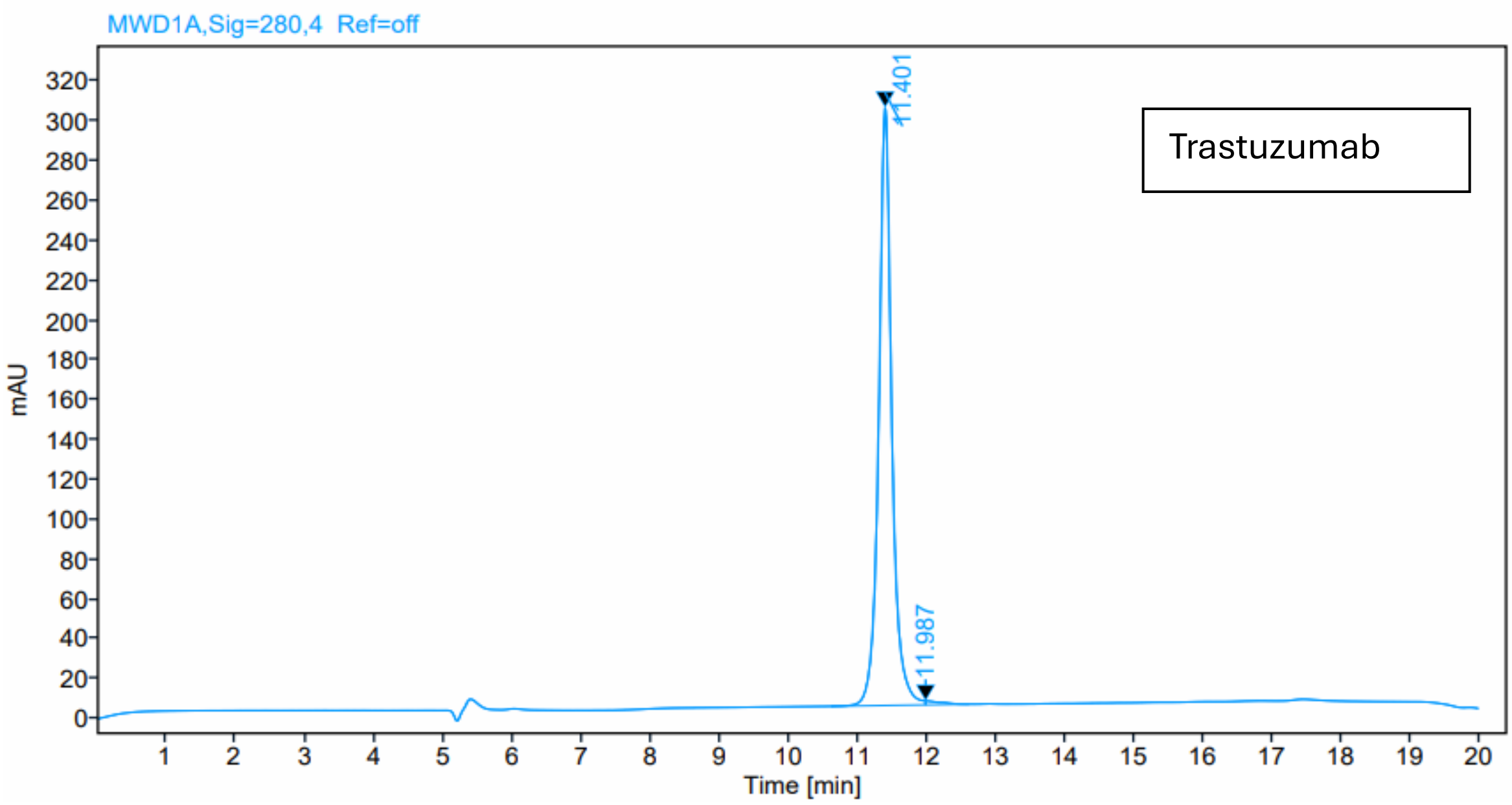

Signal: MWD1A,Sig=280,4 Ref=off

Name	RT [min]	Area	Area%	Height	Width 50% [min]
	11.4	3984.2	99.2	300.3	0.183
	12.0	30.7	0.8	2.0	0.310
	Sum	4014.8845			

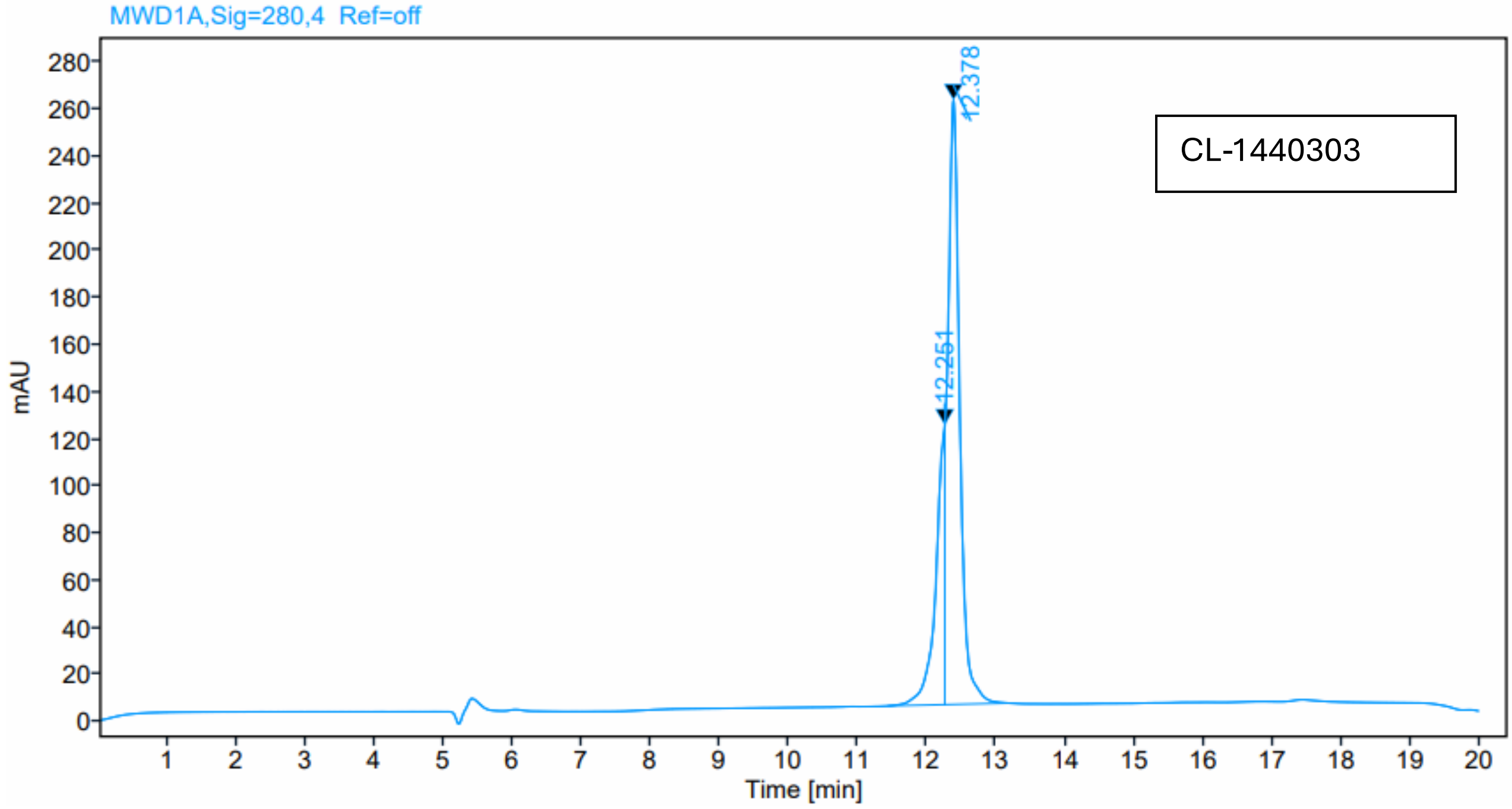

Signal: MWD1A,Sig=280,4 Ref=off

Name	RT [min]	Area	Area%	Height	Width 50% [min]
	12.3	1042.7	25.3	118.6	0.119
	12.4	3076.8	74.7	256.5	0.197
	Sum	4119.5060			

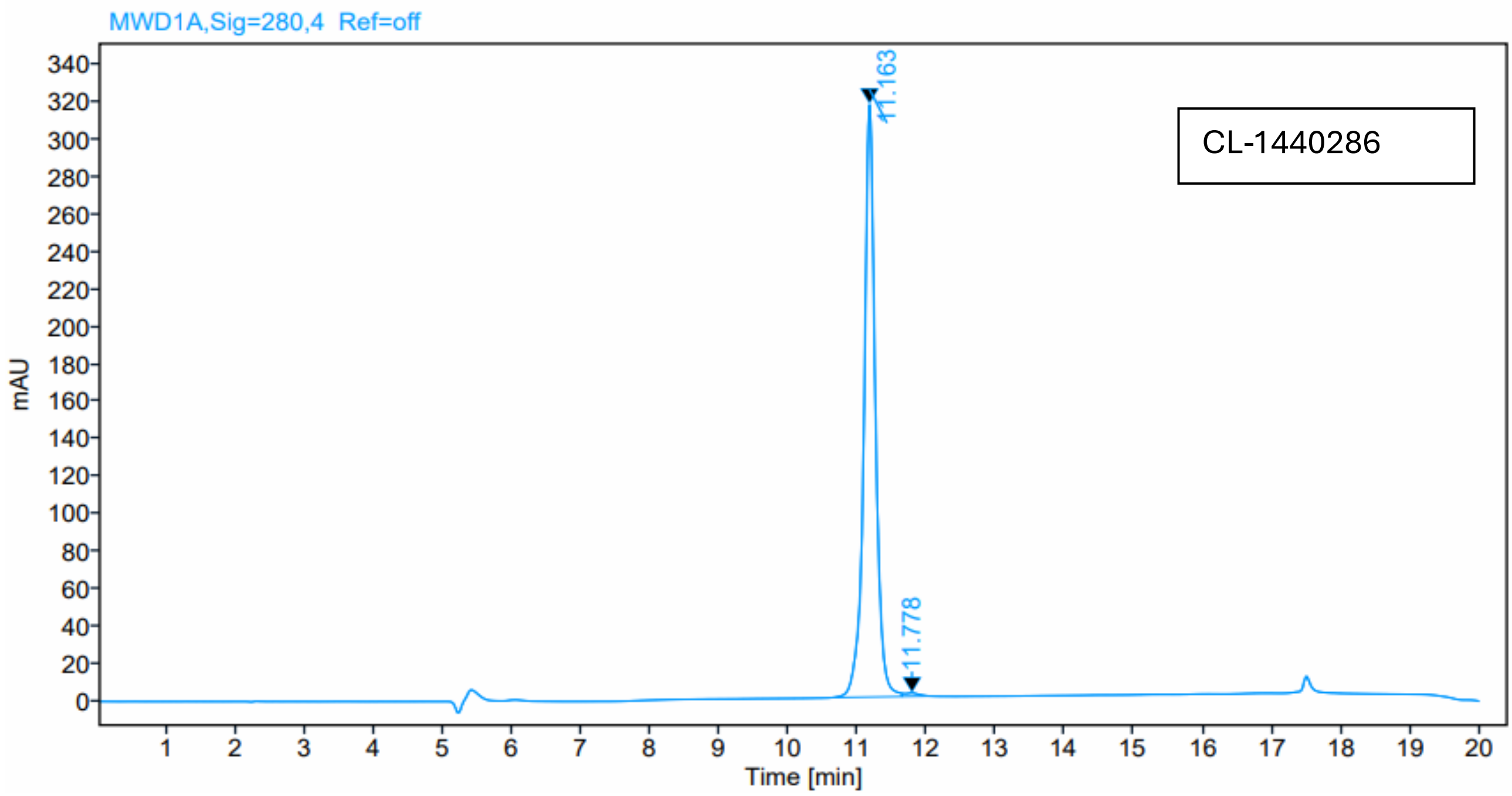

Signal: MWD1A,Sig=280,4 Ref=off

Name	RT [min]	Area	Area%	Height	Width 50% [min]
	11.2	3657.7	99.3	316.7	0.161
	11.8	27.0	0.7	1.8	0.262
	Sum	3684.6876			

Figure S6 C - aCEX

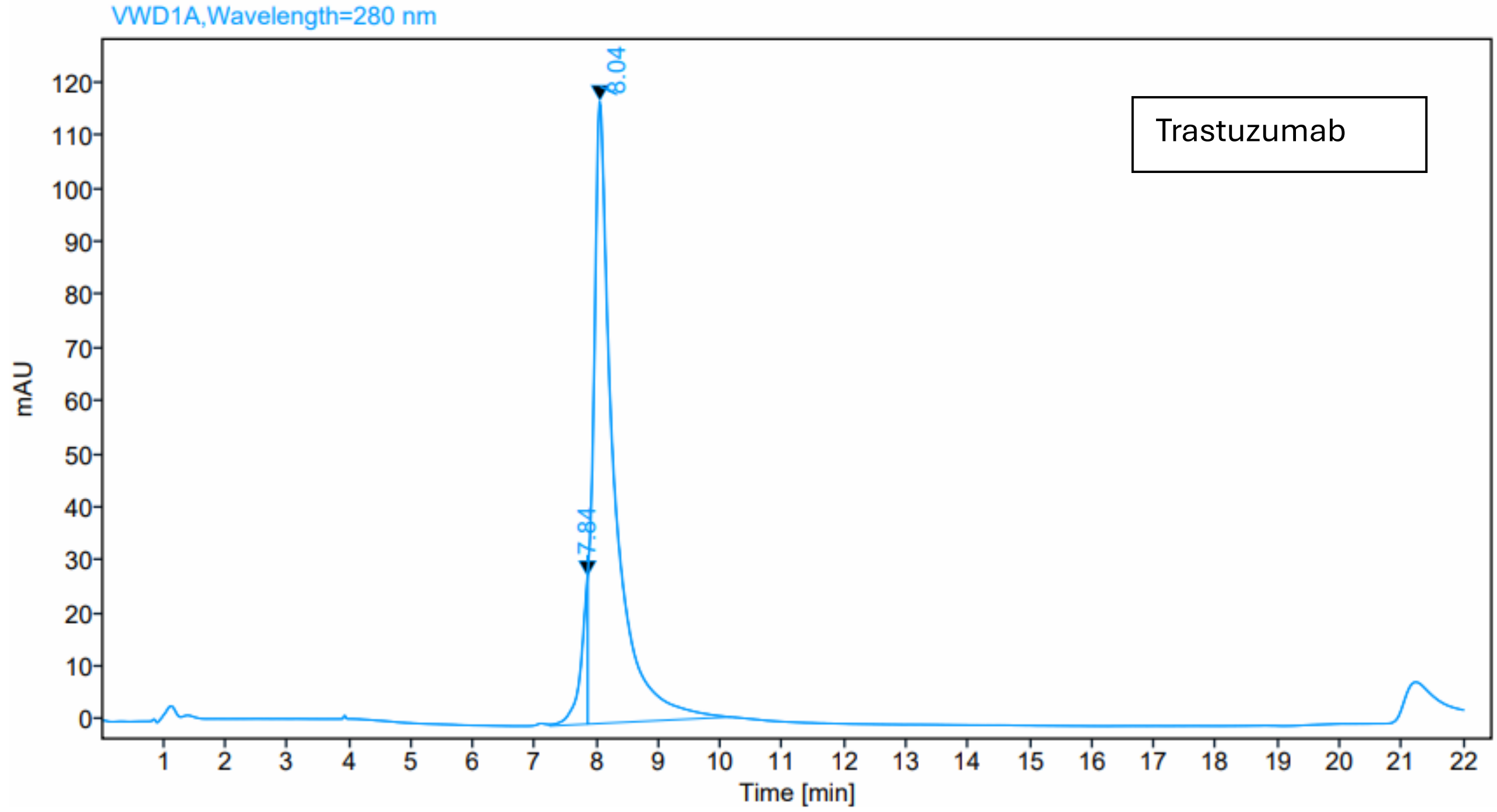

Signal: VWD1A,Wavelength=280 nm

Name	RT [min]	Area	Area%	Height	Width 50% [min]
	7.8	200.5	6.9	27.7	0.087
	8.0	2710.2	93.1	117.2	0.279
	Sum	2910.7474			

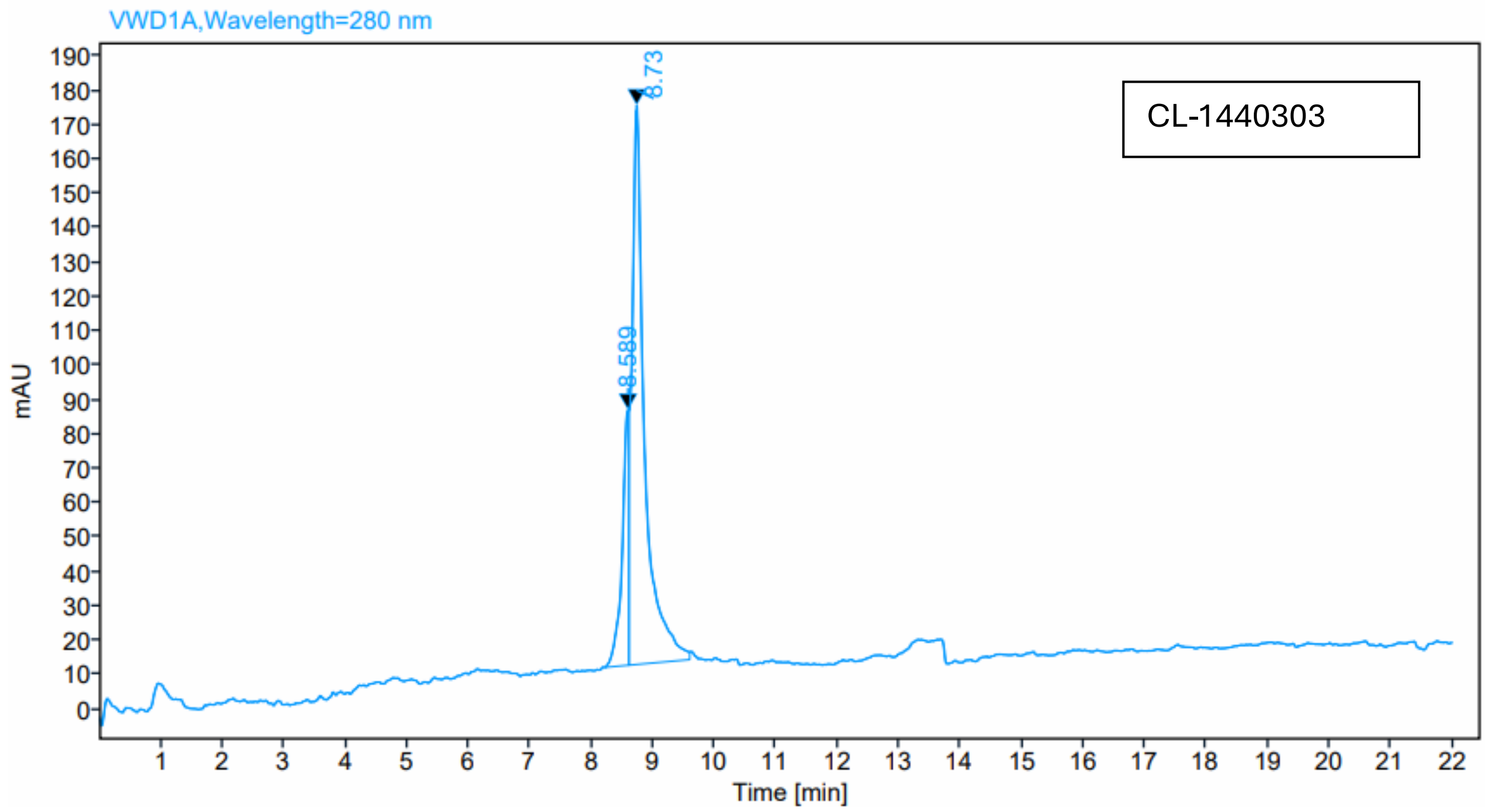

Signal: VWD1A,Wavelength=280 nm

Name	RT [min]	Area	Area%	Height	Width 50% [min]
	8.6	544.8	18.7	74.3	0.101
	8.7	2366.6	81.3	162.8	0.191
	Sum	2911.3905			

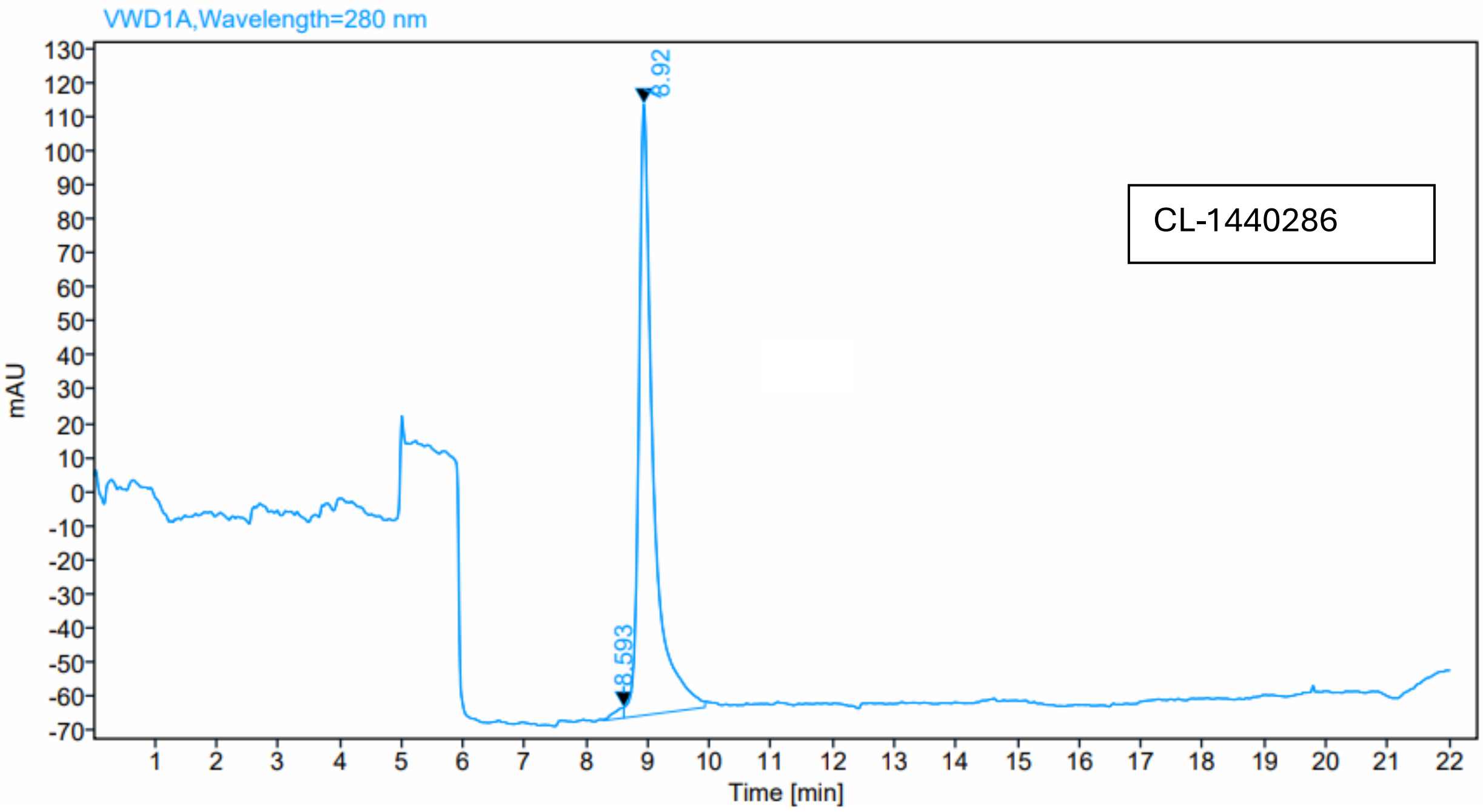

Signal: VWD1A,Wavelength=280 nm

Name	RT [min]	Area	Area%	Height	Width 50% [min]
	8.6	35.0	1.2	3.0	0.158
	8.9	2940.3	98.8	179.7	0.206
	Sum	2975.2686			

Figure S6 C - cE-SDS reducing

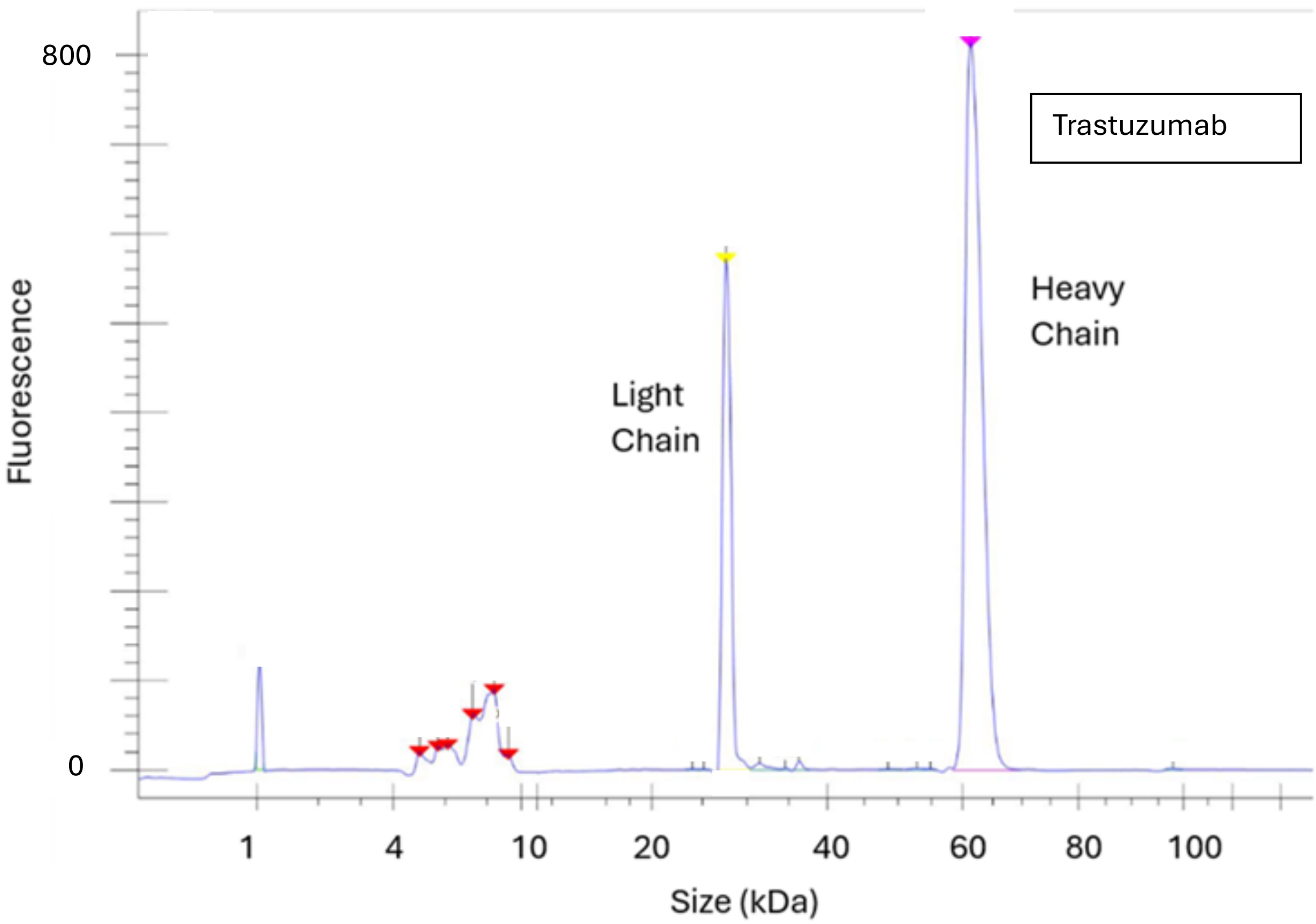

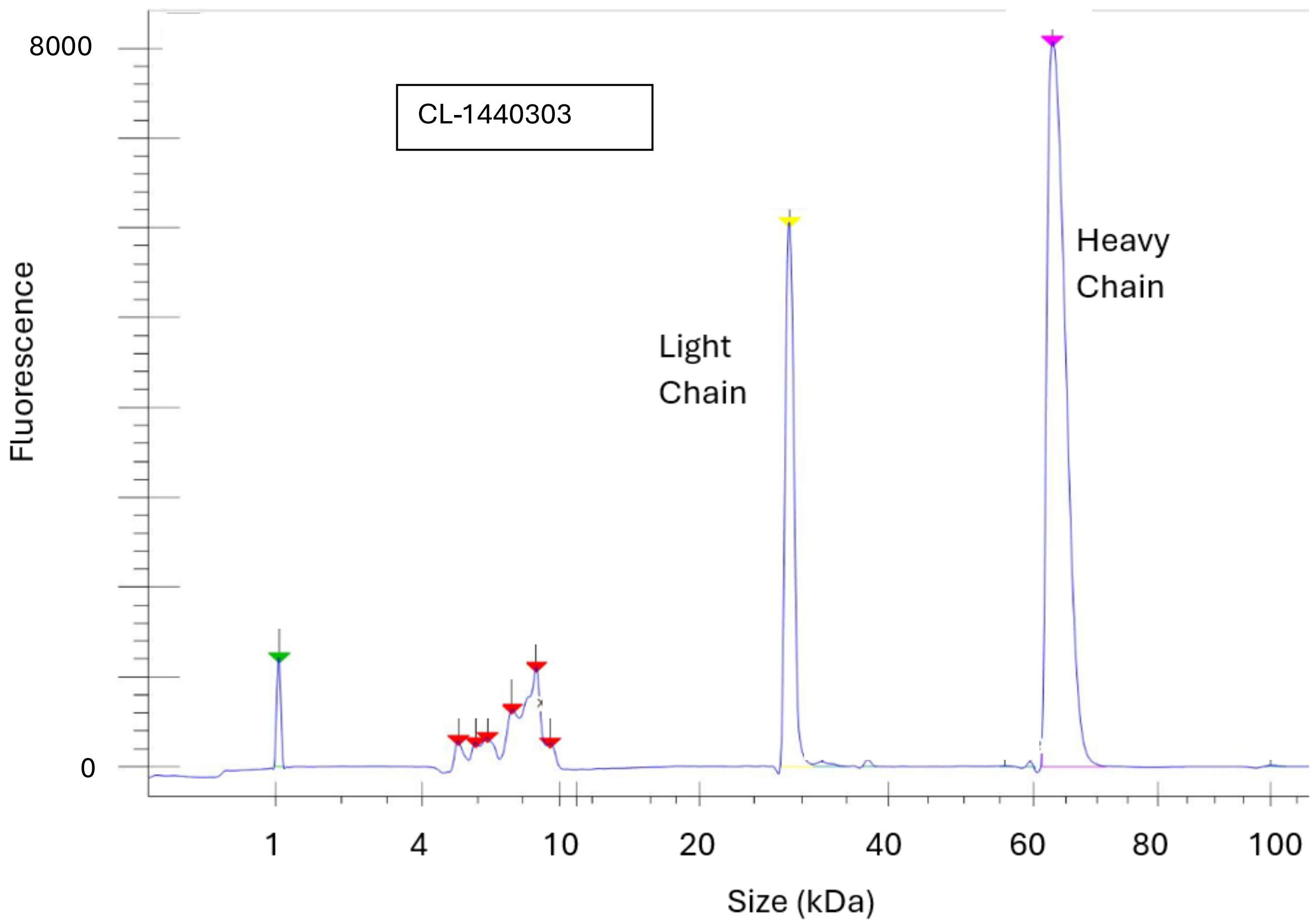
CL-1440303
8000
0
Fluorescence
Light
Chain
Heavy
Chain
1
4
10
20
40
60
80
100
Size (kDa)

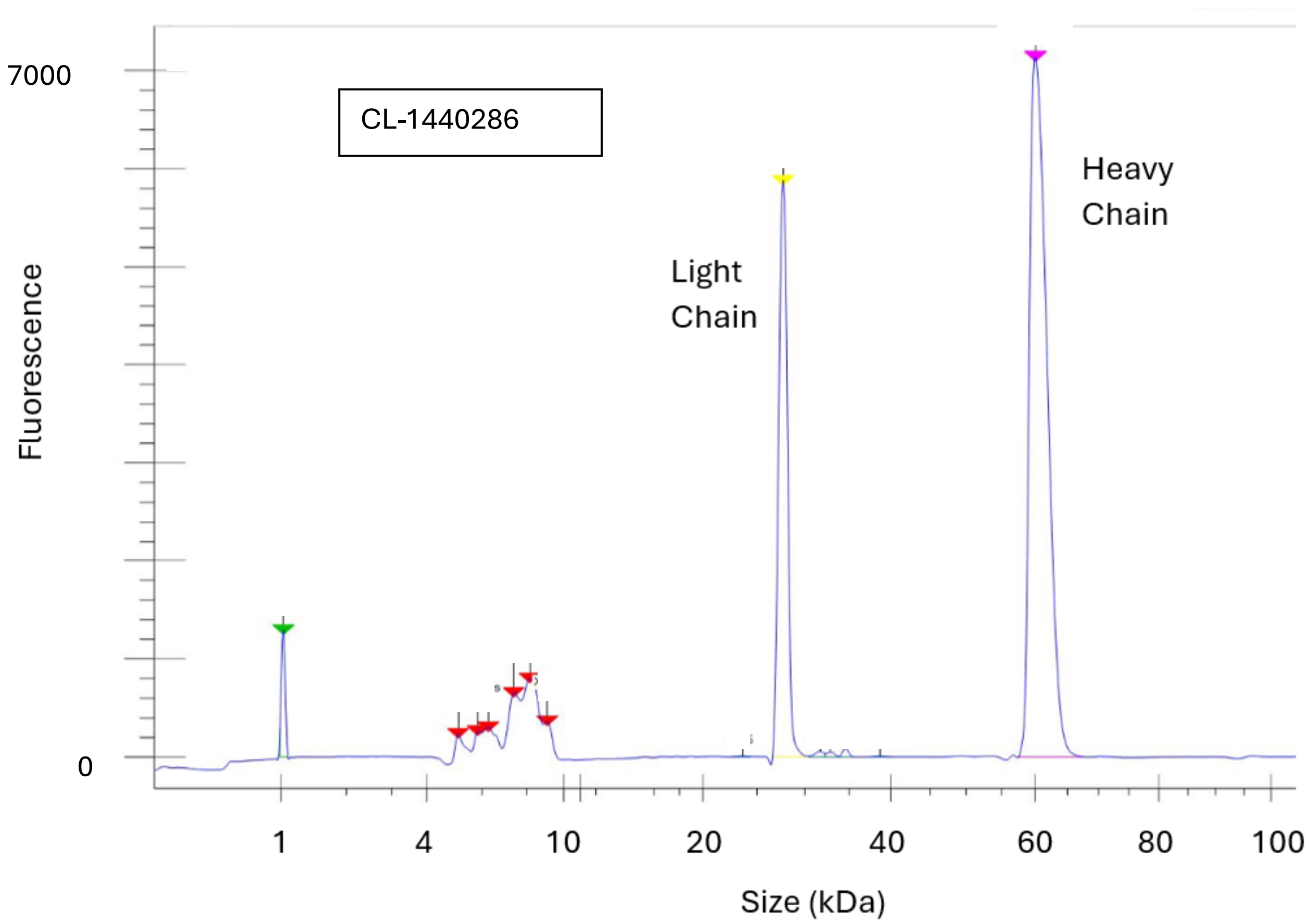
CL-1440286
7000
0
Fluorescence
Light
Chain
Heavy
Chain
1
4
10
20
40
60
80
100
Size (kDa)

Figure S6 C

- cE-SDS non-reducing

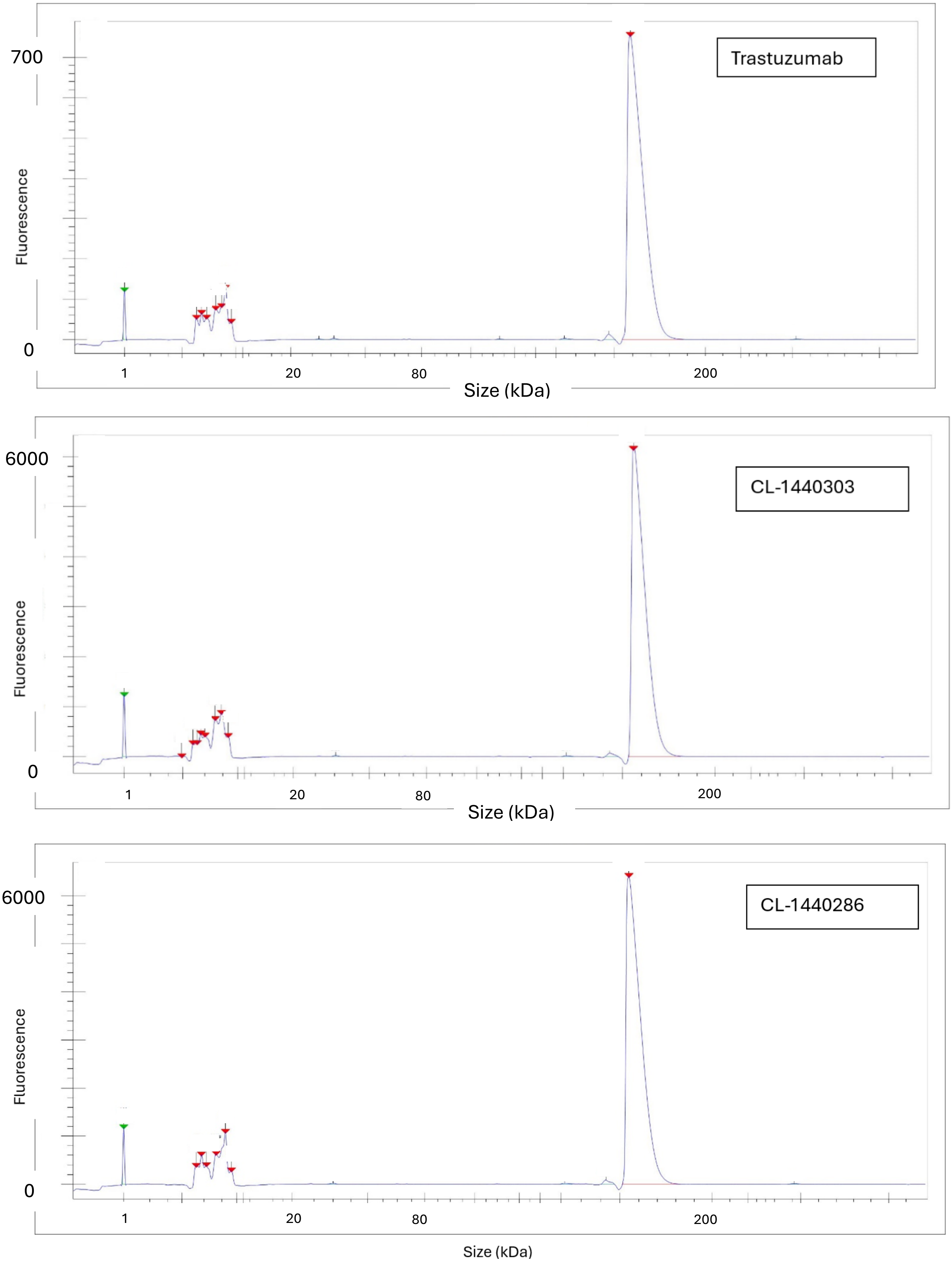

Supplement: Supplementary file 1 [file SupplementaryFile1.pdf]
